
JOURNAL INFORMATION
==============================
NLM Title Abbreviation: Innov Surg Sci
Journal ID: Innov Surg Sci
NLM Journal ID: 101708165
Journal ID: iss

Innovative Surgical Sciences

EISSN: 2364-7485
Publisher: De Gruyter

ARTICLE INFORMATION
==============================
PMCID: PMC13068495
DOI: 10.1515/iss-2026-2001
Article ID: iss-2026-2001
Article version: 1
Subjects: Article

1. Surgery (also interdisciplinary)

Electronic publication date: 2026 Apr 10
Publication date: 2026 Apr
Volume: 11
Issue: Suppl1
First page: 1
Last page: 76
Copyright: © 2026 by Walter de Gruyter Berlin/Boston
Copyright year: 2026
Copyright holder: De Gruyter
License: This work is licensed under the Creative Commons Attribution 4.0 International License.
License URL: https://creativecommons.org/licenses/by/4.0/

Page count: 76

==============================

DE GRUYTER BRILL

**Structural Work-Family Barriers in Surgery: A Gendered Nationwide Survey in Germany**
ID: E-DCK-22

**Chirurgie (auch interdisziplinär)/ Surgery (also interdisciplinary)**
**Arbeitszeitmodelle / Working Hour Models**

Valerie Nottberg[1], Lilly Klingelhöfer[1], Natasha Schweitzer[1], Sarah Keller[2], Thilo Hackert[1], Isabel Molwitz[3], Jan Bardenhagen[1], Asmus Heumann[1]

[1]Universitätsklinikum Hamburg-Eppendorf, Klinik für Allgemein-, Viszeral- und Thoraxchirurgie, Hamburg, Deutschland, [2]Charité Universitaetsmedizin Berlin, Diagnostische und interventionelle Radiologie und Nuklearmedizin, Berlin, Deutschland, [3]Universitätsklinikum Hamburg-Eppendorf, Klinik und Poliklinik für Diagnostische und Interventionelle Radiologie und Nuklearmedizin, Hamburg, Deutschland

**Background:**
Despite increasing numbers of women entering surgical training, gender gaps in advancement and leadership persist. Family responsibilities are frequently cited as career barriers but are rarely quantified across specialties and career stages. This study examines gender differences in family planning, caregiving, work patterns, and perceived institutional support among surgeons in Germany.

**Material and methods:**
We conducted a nationwide cross-sectional online survey (June–September 2024) of 432 surgeons and residents across multiple surgical disciplines. A validated 68-item instrument captured demographics, employment patterns, family planning, childcare arrangements, and perceived career barriers. Analyses included descriptive statistics, chi-square and t-tests, and regression models adjusted for age and institution; women were coded as 1 (men=0). Ethical approval was obtained from the ethics committee Hamburg. Participation was anonymous and consented.

**Results:**
Female surgeons comprised 73% of respondents and were less frequently employed at university hospitals than men (53.5% vs. 88.8%; OR 0.11, 95% CI 0.05-0.21). Women were far more likely to work part-time (25.5% vs. 5.4%; OR 12.03, 95% CI 4.19-50.87) and to experience prolonged specialization (47.1% vs. 31.2%; OR 1.74, 95% CI 1.03-2.96), with extensions linked to parental leave (OR 3.04, 95% CI 1.32-8.26). Nearly half of women postponed childbearing for career reasons (46.2% vs. 14.7%; OR 4.72, 95% CI 2.34-10.23), were more often childless (53.7% vs. 25.6%), and had fewer children ($\beta$ = -0.55). During pregnancy, 46.4% of women reported reassignment to non-surgical duties. Parenthood was perceived to hinder careers by 82.5% of women versus 11.1% of men (OR 11.22, 95% CI 4.77-30.0). Domestic and caregiving loads were uneven: women reported more weekly hours of unpaid work ($\beta$ = 5.49), undertook most family logistics (OR 37.61, 95% CI 13.58-135.53), and were more likely to stay home when a child was ill (OR 6.80, 95% CI 2.21-29.83). Public childcare was used more often by female surgeons (OR 5.83, 95% CI 2.89-12.22), and one-third of women would increase working hours with reliable childcare (OR 5.76, 95% CI 2.16-18.38). Emotional (OR 2.61, 95% CI 1.39-5.20) and physical exhaustion (OR 2.32, 95% CI 1.32-4.17) were reported more frequently by women. Female surgeons expressed stronger preferences for flexible work arrangements (OR 2.29, 95% CI 1.30-4.14), job-sharing

Open Access. © 2026 The Author(s), published by De Gruyter. This work is licensed under the Creative Commons Attribution 4.0 International License.

(OR 2.40, 95% CI 1.28–4.73), flatter hierarchies (OR 1.43, 95% CI 0.72-3.03) and gender-balanced teams (OR 6.12, 95% CI 2.9-14.63) compared to their male counterparts (Fig. 1).

**Conclusion:**
Work-family reconciliation remains structurally gendered in Germany. Family responsibilities disproportionately affect female surgeons' careers, contributing to slower advancement and increased physical and mental exhaustion. Institutional reforms such as flexible and predictable work models, accessible childcare, transparent promotion criteria that account for family-related interruptions, and mentorship are critical for retaining female talent and achieving equitable leadership pathways.

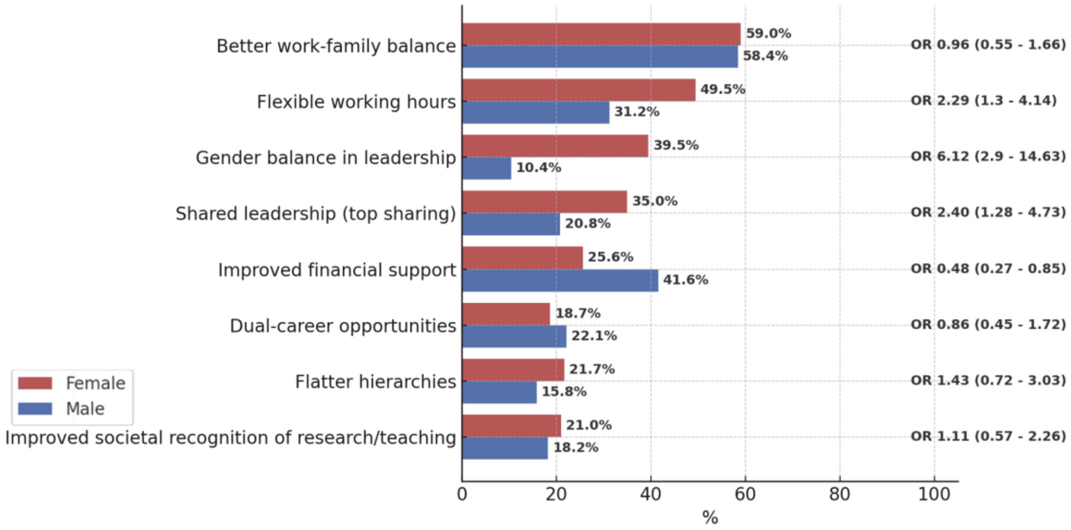

DE GRUYTER BRILL

### Client-Provider Ratio and Maternal Mortality in the OBGYN Hospitals in Kabul, Afghanistan
ID: E-DCK-431

**Chirurgie (auch interdisziplinär)/ Surgery (also interdisciplinary)**
**Auswirkungen der Gesundheitsreform / Impact of the Healthcare Reform**

Najmussama Shefajo[1]

[1]The Afghan Society of Obstetricians and Gynecologists (AFSOG), Kabul, Afghanistan

**Background:**
The World Health Report 2006 highlighted a global health workforce crisis, projecting a shortage of 17 million health workers by 2030. Afghanistan faces one of the lowest health workforce densities in the Eastern Mediterranean Region, with only 4.6 doctors, nurses, and midwives per 10,000 people far below the critical threshold of 23 per 10,000. This shortage has become particularly severe in recent years, further exacerbated by insecurity, cultural and socio-economic barriers, and regulatory challenges, resulting in pronounced gender and geographic disparities in access to care. This study explores the association between client–provider ratios and maternal mortality rates (MMR) across major hospitals in Kabul, Afghanistan.

**Material and methods:**
A multi-site demographic survey was conducted from January to March 2025 across both governmental and private obstetrics and gynecology hospitals in Kabul. Data on deliveries and maternal deaths were collected to evaluate healthcare service availability and the density of female healthcare providers. Regression analysis was used to assess the relationship between maternal mortality and client–provider ratios.

**Results:**
A total 30,000 deliveries were recorded during the study period. The ratio of births to one midwife in the maternity department was 116:1. On average, there were 441 births per specialist of obstetrics and gynecology. Regression analysis demonstrated a significant inverse association between maternal deaths and the availability of healthcare providers, indicating that higher client–provider ratios correlate with increased maternal mortality risk.

**Conclusion:**
This study highlights that high client–provider ratios in Kabul’s obstetric and gynecological hospitals are closely linked with increased maternal mortality rates. Limited numbers of trained female healthcare providers remain a key challenge, partly influenced by ongoing barriers to education and professional training opportunities for girls and women. Strengthening the maternal health workforce, ensuring equitable distribution of skilled providers, and creating supportive pathways for women’s education and medical training are essential steps toward improving maternal health outcomes in Afghanistan.

DE GRUYTER BRILL

**Risk factors of lymphatic fistulas and postoperative management in oncological lymphadenectomy**
ID: E-DCK-180

**Chirurgie (auch interdisziplinär)/ Surgery (also interdisciplinary)**
**Chirurgische Onkologie / Surgical Oncology**

Lena Scheibert[1], Stefan M. Brunner[1], Markus Götz[1], Florian Bitterer[2], Susanne Gottsauner[3]

[1]University hospital Regensburg, Klinik und Poliklinik für Chirurgie, Regensburg, Deutschland, [2]Artos Praxisgemeinschaft, Allgmeinchirurgie, Regensburg, Deutschland, [3]University hospital Regensburg, Regensburg, Deutschland

**Background:**
Surgical lymphadenectomy is still commonly performed as diagnostics and therapeutical treatment of metastasis in malignant melanoma, breast cancer, Lymphoma, and others. The high morbidity and complication rate of up to 72.9%, including lymphatic leaks, -fistulas, lymphoceles and lymphatic oedema is still an obstacle in lymph node surgery.

**Material and methods:**
Herein we analyze risk factors for the occurrence of lymphatic fistulas after axillary and inguinal lymphadenectomy in a cohort of 278 patients. 90.7% of our patients were diagnosed by malignant melanoma. 55.4% of the patients were undergoing axillary and 44.6% inguinal lymphadenectomies between 2012 and 2022.

**Results:**
In total 115 patients developed a postoperative lymphatic fistula (41.4% of the cohort). Lymphatic fistulas occurred significantly more often after inguinal lymphadenectomy compared to axillary lymphadenectomy ($p = 0.038$, Fisher-exact). The extend of the surgery was relevant for the postoperative outcome and morbidity. Thus, patients after total lymph node dissection (TLND) showed a 1.9-times higher risk to develop lymphatic fistulas compared to those who received completion lymph node dissection (CLND) or single lymph node extirpation (SLE) ($p = 0.018$). Accordingly, patients after small procedures like SLE had the lowest risk to develop lymphatic leakage complications with an OR of only 0.26 ($p = 0.045$). In inguinal lymphadenectomy, surgical crossectomy was detected as risk factor for the occurrence of postoperative lymphatic fistulas with a OR of 1.8 but failed to reach significance within our cohort ($n = 124$; $p = 0.142$). No clearly protective tendency between usage of different intraoperative devices could be seen. Patients with postoperative lymphatic fistula showed significantly more often wound healing disorders (20.0% versus 10.4%, $p = 0.036$) and wound infections (9.6% versus 3.1%; $p = 0.034$) had a longer hospitalization (median 12 versus 7 days, $p < 0.001$) and obtained significantly more often radiotherapy (68.7% versus 37.43%, $p < 0.001$).

**Conclusion:**
The high morbidity through surgical lymph node metastasis treatment meets the need for critical indications, especially in malignant melanoma with its established alterative biopharmacological treatment options. Further, neoadjuvant therapies should be considered to reduce tumor mass and thereby the extent of lymphadenectomy.

DE GRUYTER BRILL

**“Standard Operating Procedure“ (SOP) - Pain Therapy in Various Tumor Manifestations & Constellations**
ID: E-DCK-230

**Chirurgie (auch interdisziplinär)/ Surgery (also interdisciplinary)**
**Chirurgische Onkologie / Surgical Oncology**

Michael Brinkers[1], Giselher Pfau[1], Jana Bergner[1], Frank Meyer[2]

[1]Otto-von-Guericke-Universität mit Universitätsklinikum, Klinik für Anästhesiologie und Intensivtherapie, Schmerzambulanz, Magdeburg, Deutschland, [2]Otto-von-Guericke-Universität mit Universitätsklinikum, Klinik für Allgemein-, Viszeral-, Gefäß- und Transplantationschirurgie, Magdeburg, Deutschland

**Background:**
Pain therapy has obtained a high importance in clinical practice in the meantime and has become indispensable, in particular, with regard to a sufficient perioperative/-interventional case management but also related to a certain approach, specific (problematic) diagnoses, syndromes and special symptomatologies.
*Aim*: To provide a prompt and standardized access to a competently developed compact “Standard Operating Procedure“ (SOP) on interdisciplinary pain therapy in tumor patients with a broad profile of (possible) diagnoses at a subspecialized medical center based on **i)** experiences from long-term clinical practice and **ii)** current references from the scientific medical literature (not to substitute completely earlier publications and text books).

**Material and methods:**
Narrative short overview on the “SOP – Pain Therapy in tumor diseases / patients“ (provided in the up-coming presentation)

**Results:**
(corner points): - In case of tumor-associated pain, opioids represent the primarily considered group of drugs for an efficient medication.
- Medication with opioids is limited by hepatic and renal insufficiency, type of opioid (e.g., Tapentadol‘s effect is not sufficient), further contraindications (e.g., MST in chronic obstructive pulmonary disease [COPD]) and mode of possible drug administration (patients cannot swallow or in lack of consciousness).
- In patients with tumor-associated pain, it needs to be asked for anxiety.
*Discussion*: A couple of years ago, the interdisciplinary working group at the University Hospital of Magdeburg (Germany) created SOP’s for postoperative pain therapy in general and abdominal surgery followed by vascular surgery / medicine in 2024.

**Conclusion:**
Tumor-associated pain belongs to the type of pain (in addition to birth pang and postoperative pain), which can be approached best.

This represents a call to action. It comprises consequent, adequate drug administration with sufficient dosage according to the WHO scheme (WHO I actually not necessary), psychotropics and drug-free procedures.

DE GRUYTER BRILL

### Fusion Imaging in Retroperitoneal Sarcoma: Correlating Tumor, Resection Margin and Local Recurrence

ID: E-DCK-293

**Chirurgie (auch interdisziplinär)/ Surgery (also interdisciplinary)**
**Chirurgische Onkologie / Surgical Oncology**

Charlotte Gündisch[1], Dominik Nörenberg[2], Helene Weigl[1], Franka Menge[1], Melissa Harbrücker[1], Christoph Reißfelder[3], Miriam Eckel[4], Anna Simeonova-Chergou[4], Peter Hohenberger[1], Jens Jakob[1], Madelaine Hettler[1]

[1]University medical center and medical faculty Mannheim, University of Heidelberg, Department of surgery, sarcoma unit, Mannheim, Deutschland, [2]University Medical Center Mannheim, University of Heidelberg, Department of Radiology and Nuclear Medicine, Mannheim, Deutschland, [3]University Medical center and medical faculty Mannheim, University of Heidelberg, Department of Surgery, Mannheim, Deutschland, [4]University Medical Center Mannheim, University of Heidelberg, Department of Radiation Oncology, Mannheim, Deutschland

**Background:**
Retroperitoneal soft tissue sarcomas (RPS) are rare tumors. The mainstay of treatment is complete resection. Neoadjuvant radiotherapy has shown a significant benefit only for selected subtypes. The effect of neoadjuvant chemotherapy is currently being evaluated in the STRASS II trial. In expert centers, complete is achieved by multivisceral resection in up to 95% of all patients and the rate of local recurrence (LR) is approximately 30%. (Gronchi et al. 2016). Since local recurrence is a rather a frequent event even after multimodal treatment and multivisceral resections at expert centers, the analysis of recurrent cases is of utmost importance. Fusion imaging with detection of the exact anatomical correlation of primary tumors and local recurrence taking into account the region of the closest resection margin may contribute to improved treatment allocation and sarcoma surgery.

**Material and methods:**
In this pilot study, we retrospectively analyzed 10 patients with abdominal LR after RPS resection at the sarcoma center Mannheim. CT/MRI datasets of primary tumor (PT) and LR were fused using Velocity (Version 4.1, Varian Medical Systems). Registration was performed in a stepwise manner. First, rigid auto-registration was applied to achieve coarse alignment. Manual editing of rigid registrations was used, if necessary, to optimize alignment of anatomical landmarks. Deformable registration using a B-spline–based algorithm was performed when rigid alignment was insufficient. Recurrence sites were classified as “within”, “adjacent”, or “distant from” the PT volume. In addition, we analysed the anatomical correlation of the closed resection margin (according to the pathology report) and the local recurrence. Imaging data were analyzed by a medical student (CG) and a surgical resident (MH) and validated by an experienced radiologist (DN).

**Results:**
An accurate fusion imaging was feasible in all cases. In 5/10 (50%) cases, LR was located within the anatomical boundaries of the PT; in 4/10 (40%), LR was adjacent; and in 1/10 (10%), LR was distant. In 2 cases LR occurred at sites where resection margins were histologically incomplete, in 2 cases at sites corresponding to narrow resection margins (≤ 0,1 cm), and in 4 patients at sites unrelated to either incomplete or narrow margins. In 2 cases the margins were not classified in the pathological report. Interestingly in one patient with an R1 resection at the caudal margin, the recurrence occurred proximally.

**Conclusion:**
Fusion imaging holds the potential to provide meaningful insights into recurrence patterns of RPS, which were associated with resection margins in half of the cases in this analysis. If validated in larger cohorts, this technique could refine recurrence risk stratification and inform both surgical planning and (neo-)adjuvant treatment strategies for RPS.

DE GRUYTER BRILL

### Delaying Major Hepatectomy After Chemotherapy Reduces Morbidity in Colorectal Liver Metastases

ID: E-DCK-323

**Chirurgie (auch interdisziplinär)/ Surgery (also interdisciplinary)**
**Chirurgische Onkologie / Surgical Oncology**

Esther Giehl-Brown[1], Rajan Nikbakhsh[1], Ana Mansourkiaei[1], Laila Jötten[1], Bruno Köhler[1], Thomas Longerich[1], Zoltan Czigany[1], Bo Kong[1], Arianeb Mehrabi[1], Mw Büchler[2], Mohammed Al-Saeedi[1], Christoph Kahlert[1]

[1]Universitätsklinikum Heidelberg, Heidelberg, Deutschland, [2]Botton-Champalimaud Pancreatic Cancer Center, Lissabon, Portugal

### Background:

The optimal timing of resection for colorectal liver metastases (CRLM) after systemic chemotherapy remains uncertain. This study evaluated the impact of time-to-surgery (TTS) on postoperative morbidity and oncologic outcomes.

### Material and methods:

In this retrospective cohort (2018–2022) from a high-volume hepatobiliary center in Germany, 159 patients underwent hepatic resection for CRLM following chemotherapy. Patients were stratified by TTS ≤41 versus ≥42 days. The primary endpoint was clinically meaningful morbidity (Comprehensive Complication Index ≥ 30). Secondary endpoints included liver-specific recurrence-free survival (RFS) and overall survival (OS). Multivariable regression, Cox proportional-hazards models, and prespecified subgroup analyses were performed.

### Results:

Clinically meaningful morbidity occurred in 48.7% of patients with shorter TTS versus 31.3% with longer TTS (P = 0.023). TTS ≥ 42 days was associated with a 65% reduction in adjusted odds of morbidity (OR 0.35; 95% CI 0.13–0.98; P = 0.045). In major hepatectomy, TTS ≥ 42 days reduced adjusted odds by 92% (OR 0.078; 95% CI 0.011–0.573; P = 0.012). TTS did not independently predict RFS (HR 0.85; 95% CI 0.54–1.34; P = 0.49), and OS was comparable (log-rank P = 0.530).

### Conclusion:

Delaying surgery to ≥6 weeks after chemotherapy significantly decreased major morbidity without compromising early oncologic outcomes. These findings support a prolonged interval before complex resections, warranting prospective validation.

**Limb preservation in an humanitarian orthoplastic pilot project**
ID: E-DCK-386

**Chirurgie (auch interdisziplinär)/ Surgery (also interdisciplinary)**
**Extremitätenrekonstruktion / Limb Reconstruction**

Gerrit Freund[1], Simon Mayer[2], Rolf Overs-Frerker[3], Hans-Elmar Nick[3], Olaf Bach[4]

[1]UKSH, Campus Lübeck, Lübeck, Deutschland, [2]BGU Ludwigshafen, Ludwigshafen, Deutschland, [3]SAH Eschweiler, Eschweiler, Deutschland, [4]Zomba Central Hospital, Zomba, Malawi

**Background:**
In October 2024 a pilot project, representing a collaborative effort between INTERPLAST Germany e.V.,  Sektion Eschweiler and Zomba Hospital Projekt e.V., was initiated aiming at addressing orthoplastic surgical needs within a humanitarian aid.Zomba Central Hospital is one of four central hospitals of the Malawi Ministry of Health and serves approximately 4.5 million people. For this purpose, a team consisting of three plastic surgeons and three anesthetists was formed. The team consisted of two experienced specialists, two specialists in their first assignment and two doctors in further training. The mission was planned for 14 days. A second mission was done in September 2025, also for 14 days.

**Material and methods:**
Patients were preselected by an trauma team from Zomba Hospital Project e.V. which was previously on site. Preselected patients were presented in a form a extremity board according to European standards via online meetings with clinical examinations, pictures and radiographic findings.

**Results:**
A total of 149 patients with a mean age of 26,9 years (± 18,6, M = 28) were treated in both missions, 46 of whom had defects requiring coverage following open fractures, osteomyelitis, or tumors. A total of 14 pedicled muscle flaps, 7 random pattern flaps, and 7 perforator-based flaps were performed. While pedicled muscle flaps can be performed for the proximal and middle thirds, free flaps are considered the gold standard for the lower third. Due to a lack of resources, technical equipment, and experience in follow-up care, the options for microsurgical reconstruction are often limited in Southeast African countries. Besides classic muscle flaps, perforator based propeller flaps were a reliable choice for lower third defects.The initiative not only addressed immediate medical needs but also contributed to building local capacity by training healthcare professionals in advanced surgical techniques. Therefor local OR nurses and medical officers were trained by the interdisciplinary orthoplastic team. Due to the cooperation a follow up of the complex orthoplastic patients within a range of three month could be implemented. No flap loss was recorded and two patients with flap coverage needed secondary grafting after a muscle flap. In total four different flap procedures could be teached to the local trauma team.

**Conclusion:**
Results from this pilot project underscore the feasibility and efficacy of international and interdisciplinary collaborations in humanitarian medicine providing specialized care in low-resource environments, with potential for future expansion. Further evaluation and longitudinal studies are recommended to assess long-term patient outcomes and the impact on local healthcare systems. Hospitals in south-east Africa are often lacking a specialiszed team for soft tissue reconstruction furthermore specialized orthoplastic treatment is rarely available. A training of local medical teams can lead to an increased health care and, specially in the case of orthoplastic surgery, decrease the number of amputations.

DE GRUYTER BRILL

**Factors Influencing Recurrence After In-Hospital Treatment of Odontogenic-Related Infections**
ID: E-DCK-114

**Chirurgie (auch interdisziplinär)/ Surgery (also interdisciplinary)**
**Infektionschirurgie / Infection Surgery**

Mario Scheurer[1], Jasmin Engelbrecht[1], Johannes Schulze[1], Marcel Ebeling[1], Robin Kasper[1], Stefan Kist[1], Majeed Rana[1], Frank Wilde[1], Alexander Schramm[1], Andreas Sakkas[1]

[1]Universitätsklinikum Ulm, Klinik für Mund-, Kiefer- und Gesichtschirurgie, Bundeswehrkrankenhaus Ulm, Klinik für Mund-, Kiefer- und Plastische Gesichtschirurgie, Ulm, Deutschland

**Background:**
Oromaxillofacial infections of odontogenic origin are among the most common emergencies in oral and maxillofacial surgery. While effective treatment generally includes drainage, source control and antibiotics, recurrence remains a significant clinical challenge. This study aimed to identify factors associated with infection recurrence and prolonged hospital stay (LOS) following in-hospital treatment.

**Material and methods:**
A retrospective single-center study was conducted on 939 patients treated surgically or conservatively for odontogenic-related oromaxillofacial infections between 2019 and 2023. Demographic, clinical, laboratory, microbiological and treatment-related variables were analyzed. Recurrence within 6 months was defined as the primary endpoint. LOS was the secondary endpoint. Multivariable logistic regression was performed to identify independent predictors of recurrence and LOS.

**Results:**
The recurrence rate was 5.0%, with a mean LOS of 3.6 days. Independent predictors of recurrence included age ≥ 50 years, BMI ≥ 30, mandibular infection, post-extraction or post-augmentation etiology, restricted mouth opening, dysphagia, non-palpable mandibular margin, elevated inflammatory markers, polymicrobial infections, cervical drainage, lack of continuous postoperative antibiotic therapy and ICU admission (OR up to 9.1, $p < 0.05$). Longer LOS was associated with older age, higher BMI, mandibular involvement, systemic comorbidities, elevated CRP and surgery under intubation anesthesia. A structured Clinical Recurrence Risk Score was developed, integrating these predictors with good discriminatory ability (AUC = 0.80).

**Conclusion:**
This large single-centre study identifies clinical, laboratory, and treatment-related predictors of recurrence and prolonged hospitalization in odontogenic-related oromaxillofacial infections. Early recognition of high-risk patients may guide more aggressive surgical management, tailored antibiotic regimens, and intensified follow-up. Prospective multicenter validation of the proposed risk score is warranted to support standardized, evidence-based treatment protocols.

DE GRUYTER BRILL

**AI in postoperative infection detection: optimized lab test timing by surgical cluster**
ID: E-DCK-350

**Chirurgie (auch interdisziplinär)/ Surgery (also interdisciplinary)**
**Infektionschirurgie / Infection Surgery**

Yves Wintsch[1], Tobias Blatter[1], Karen Triep[2], Olga Endrich[2 3], Hugo Armando Guillen-Ramirez[1], Guido Beldi[1]

[1]Department of Visceral Surgery and Medicine, Inselspital, Bern University Hospital, University of Bern, Bern, Switzerland, Bern, Schweiz, [2]Medical Directorate, Medizincontrolling, Inselspital, University Hospital Bern, Insel Gruppe, Bern, Schweiz, [3]Department of Clinical Chemistry, Inselspital, Bern University Hospital, University of Bern, Bern, Schweiz

**Background:**
Postoperative bacterial infections remain a leading cause of morbidity across surgical specialties. Although preoperative laboratory testing is standardized, postoperative testing varies widely, leading to inconsistent detection of infections and inefficient use of resources. This study aimed to generate procedure-specific recommendations for optimal laboratory monitoring during the early postoperative period using electronic health record (EHR) data and machine learning.

**Material and methods:**
We retrospectively analysed 32,328 surgical cases at a tertiary care centre (2014–2022). Surgical procedures were grouped into seven clinically coherent clusters (thoracic, cardiac, vascular, abdominal wall, gastrointestinal, hepatobiliary, urologic) using semantic embeddings of CHOP codes. The primary endpoint was occurrence of bacterial infection, defined by curated ICD-10 codes. Random Forest models incorporated preoperative (age, sex, ASA score, comorbidities), intraoperative (surgery duration, emergency status, ICU transfer, antibiotic use), and postoperative (laboratory markers and kinetics) features. We identified laboratory tests that improved predictive performance on postoperative days (POD) 0–2 and derived day-specific thresholds for infection risk.

**Results:**
The overall infection rate was 17.2%, ranging from 9.9% (cardiac) to 26.8% (gastrointestinal). The model achieved an AUROC of 0.78 on POD 2. C-reactive protein (CRP) consistently improved prediction across all clusters and PODs. Additional markers showed cluster-specific relevance: alkaline phosphatase (cardiac, abdominal wall), creatinine (cardiac, urologic), platelets (thoracic, abdominal wall, hepatobiliary), and neutrophils (abdominal wall, hepatobiliary). Thresholds varied substantially by cluster and POD, e.g., CRP ranging from 23 mg/L in gastrointestinal surgery on POD 0 to >400 mg/L in thoracic surgery on POD 2. Incorporating kinetic features modestly improved performance, especially in cardiac surgery. Confounding by indication analysis confirmed that clinician-driven test ordering itself carried predictive signal.

**Conclusion:**

Machine learning applied to routine EHR data enables surgery-specific, day-specific laboratory testing recommendations for early infection surveillance. This approach streamlines postoperative monitoring, provides actionable diagnostic thresholds, and supports efficient use of laboratory resources. It advances toward standardized, evidence-based postoperative infection surveillance across diverse surgical fields.

DE GRUYTER BRILL

**Endoscopic-assisted treatment for subcondylar fractures using a patient-specific implant**
ID: E-DCK-106

**Chirurgie (auch interdisziplinär)/ Surgery (also interdisciplinary)**
**Klinische Studien / Clinical Trials**

Mario Scheurer[1], Johannes Schulze[2], Tobias Daut[2], Marcel Ebeling[1], Robin Kasper[2], Stefan Kist[1], Majeed Rana[3], Frank Wilde[1], Alexander Schramm[1], Andreas Sakkas[1]

[1]Universitätsklinikum Ulm, Klinik für Mund-, Kiefer- und Gesichtschirurgie, Bundeswehrkrankenhaus Ulm, Klinik für Mund-, Kiefer- und Plastische Gesichtschirurgie, Ulm, Deutschland, [2]Bundeswehrkrankenhaus Ulm, Klinik für Mund-, Kiefer- und Plastische Gesichtschirurgie, Ulm, Deutschland, [3]Universitätsklinikum Ulm, Klinik für Mund-, Kiefer- und Gesichtschirurgie, Ulm, Deutschland

**Background:**
The transoral endoscopic-assisted reduction and internal fixation (EAORIF) of mandibular subcondylar fractures avoids external scars and minimizes risk to the facial nerve, but the approach remains technically demanding. Conventional miniplate fixation often suffers from limited intraoperative visualization and unstable fragment reduction. This study introduces a novel CAD/CAM-based workflow - *the Ulm protocol* - using a template-guided, patient-specific osteosynthesis implant (PSOI) designed to facilitate precise intraoral fracture reduction and fixation.

**Material and methods:**
A multifragmentary subcondylar fracture was virtually reduced using 1 mm CT data. Based on backward planning, a PSOI and matching drill guide were CAD/CAM-designed with a “one-fit-only” configuration and manufactured by selective laser melting. Preclinical validation included in-vitro placement on 3D-printed models. In-vivo application was performed via a standardized EAORIF without transbuccal trocars, supported by intraoperative CBCT verification. Postoperative outcomes were analyzed by 3D deviation mapping of planned versus achieved fragment position and occlusal analysis at follow-up.

**Results:**
The PSOI achieved secure fragment capture and enabled accurate one-step reduction and fixation. Intraoperative CBCT confirmed high concordance with preoperative planning, with mean positional deviation of 0.7 mm. Postoperative occlusal analysis demonstrated stable, symmetric intercuspation within 12 weeks. The workflow proved feasible within < 7 days from imaging to implantation and was seamlessly integrated into the intraoral surgical approach.

**Conclusion:**
Template-guided PSOI enables predictable, precise, and minimally invasive management of subcondylar fractures through the transoral approach. This innovation may simplify the technically demanding EAORIF procedure, reduce operative complexity and enhance both functional and aesthetic outcomes. Larger clinical studies are warranted to validate the *Ulm protocol* as a future standard in maxillofacial trauma surgery.

DE GRUYTER BRILL

### 3D Accuracy Analysis of Mandibular Positioning Using PSI in Bimaxillary Orthognathic Surgery

ID: E-DCK-111

**Chirurgie (auch interdisziplinär)/ Surgery (also interdisciplinary)**
**Klinische Studien / Clinical Trials**

Mario Scheurer[1], Johannes Schulze[1], Robin Kasper[1], Tobias Daut[1], Stefan Kist[1], Majeed Rana[1], Frank Wilde[1], Alexander Schramm[1], Marcel Ebeling[1], Andreas Sakkas[1]

[1]Universitätsklinikum Ulm, Klinik für Mund-, Kiefer- und Gesichtschirurgie, Bundeswehrkrankenhaus Ulm, Klinik für Mund-, Kiefer- und Plastische Gesichtschirurgie, Ulm, Deutschland

**Background:**
Digital workflows with patient-specific drill and osteotomy guides (PSDOG) and osteosynthesis implants (PSOI) have become standard in maxillary repositioning. However, evidence on their accuracy and feasibility for mandibular repositioning in bimaxillary orthognathic surgery remains limited.

**Material and methods:**
This retrospective cohort study included 30 patients undergoing bimaxillary osteotomies (11 with mandibular PSDOG/PSOI vs. 19 with conventional sagittal split osteotomy and semi-rigid fixation). Pre- and postoperative CT datasets were analyzed. Accuracy was assessed by cephalometric landmarks and 3D evaluation (Dice similarity coefficient, Hausdorff distance and model-to-model deviation). Primary outcome was mandibular transfer accuracy; secondary outcomes included proximal segment and condylar positioning, intraoperative complications, and surgical time.

**Results:**
All procedures were successfully completed. Guided mandibular repositioning showed comparable overall accuracy to conventional methods, with a trend toward reduced maximal deviations at the left ascending ramus ($p = 0.06$). Cephalometric analysis revealed significant vertical undercorrections and lateral deviations of proximal segments, particularly at the condyles. Rates of iatrogenic inferior alveolar nerve injuries and “bad splits” were lower in the guided group, though differences were not statistically significant. Surgical duration was similar between groups ($p = 0.2$).

**Conclusion:**
Fully digital, splint-free mandibular repositioning using PSDOG and PSOI is feasible and reproducible in a clinical training environment. While precise control of proximal segments, especially condylar positioning, remains limited, template-guided workflows offer reliable transfer of preoperative planning with reduced complication rates. These findings highlight the potential of patient-specific solutions to further improve precision and safety in orthognathic surgery.

DE GRUYTER BRILL

### CAD/CAM-Guided Drill and Patient-Specific Implants for Primary Treatment of ZMO-Complex Fractures

ID: E-DCK-116

**Chirurgie (auch interdisziplinär)/ Surgery (also interdisciplinary)**
**Klinische Studien / Clinical Trials**

Mario Scheurer[1], Johannes Schulze[1], Robin Kasper[1], Tobias Daut[1], Stefan Kist[1], Majeed Rana[1], Frank Wilde[1], Alexander Schramm[1], Andreas Sakkas[1], Marcel Ebeling[1]

[1]Universitätsklinikum Ulm, Klinik für Mund-, Kiefer- und Gesichtschirurgie, Bundeswehrkrankenhaus Ulm, Klinik für Mund-, Kiefer- und Plastische Gesichtschirurgie, Ulm, Deutschland

**Background:**
Fractures of the zygomaticomaxillary-orbital (ZMO) complex represent a particular challenge in trauma surgery due to the complex anatomy and the high functional and aesthetic demands. Conventional manual repositioning techniques are often associated with inconsistent outcomes and revision surgeries.

**Material and methods:**
We present a fully digital treatment concept based on virtual fracture repositioning and CAD/CAM-assisted fabrication of patient-specific osteosynthesis plates and innovative drill templates. The implants were manufactured using selective laser melting. Intraoral and transconjunctival approaches were employed, with additional intraoperative 3D imaging for positional verification.

**Results:**
A highly precise anatomical reconstruction was achieved, with nearly perfect congruence between the virtual planning and the postoperative outcome. Of note is the “one-position-fits-only” design of the patient-specific orbital implants (PSI), which allows for definite intraoperative placement. Improved interdisciplinary workflows resulted in reduced planning and production times (< 7 days) as well as seamless intraoperative application.

**Conclusion:**
The results demonstrate both the technical feasibility and the clinical benefit of a fully digital, patient-specific approach for the primary management of complex midfacial fractures. This concept enables reproducible precision, reduced operating times, and improved functional and aesthetic outcomes, and may represent a paradigm shift in maxillofacial trauma surgery.

DE GRUYTER BRILL

**Sensor-controlled, step-by-step tracking of rehabilitation progress after ACL surgery**
ID: E-DCK-439

**Chirurgie (auch interdisziplinär)/ Surgery (also interdisciplinary)**
**Klinische Studien / Clinical Trials**

Andreas Kopf[1], Hermann Bail[1], Markus Geßlein[1]

[1]Klinik für Orthopädie und Unfallchirurgie, Paracelsus Medizinische Privatuniversität Nürnberg, Nürnberg, Deutschland

**Background:**
Postoperative rehabilitation following arthroscopic knee surgery, particularly anterior cruciate ligament reconstruction, is essential for successful treatment. Despite numerous recommendations, there are still no standardized measures for structured early rehabilitation. Digital medical devices (DMDs), such as Orthelligent from OPED, are increasingly being used to supplement traditional physical therapy in order to increase training adherence and objectively measure rehabilitation progress in stages. The aim of the study is to investigate factors influencing different phases of rehabilitation using routine data from the Orthelligent system. Rehabilitation outcomes after anterior cruciate ligament injury (isolated and with accompanying injuries) were examined in terms of functional development, pain progression, and influencing factors. Furthermore, the study analyzed in which rehabilitation phase (phases 0-4) the greatest functional progress was achieved and which factors influence functional progression as measured by the Limb Symmetry Index (LSI).

**Material and methods:**
In a retrospective cohort analysis, anonymous post-operative test and exercise data from 335 patients following ACL replacement were evaluated. Functional performance was assessed using the LSI index and pain was assessed using the VAS scale. A generalized linear model (GLM) using generalized estimating equations (GEE) was applied to examine progress across the rehabilitation phases. The significance of the influencing factors was tested using the Wald chi-square test. For further analysis, pairwise comparisons of the estimated marginal means of the rehabilitation phases were performed. Group comparisons of pain scores were performed using the chi-square test.

**Results:**
The evaluation of the LSI values showed significant functional progress during rehabilitation phases 0-2 ($0.64 \pm 0.02$ vs. $0.81 \pm 0.01$; $p < 0.001$). It was also shown that function, as measured by the LSI, was positively associated with the time elapsed since surgery ($p = 0.034$). Conversely, pain (measured using the VAS scale) had a significant negative impact on functional performance ($p < 0.001$). Height and body weight, on the other hand, had no significant influence. With regard to pain distribution, 87 patients (20.6%) reported values above 5 on the VAS scale. Compared to patients with lower pain scores (VAS ≤ 5), there were no significant differences in gender, weight, or age distribution.

**Conclusion:**

This study demonstrates that digital rehabilitation monitoring can reliably reflect patient progress after ACL reconstruction. The early postoperative period (first 3 months) is critical for functional gains, highlighting the need for individualized, pain-sensitive rehabilitation strategies.

DE GRUYTER BRILL

**Impact of Implementation of new Electronic Health Record System on Surgical Patient Safety**
ID: E-DCK-476

**Chirurgie (auch interdisziplinär)/ Surgery (also interdisciplinary)**
**Krankenhausreform - Krankenhaus der Zukunft / Hospital Reform – Hospital of the Future**

Jonas Fischer[1], David Rösli[1], Daniel Sanchez-Taltavull[1], Vanessa Banz[1], Guido Beldi[1], Daniel Candinas[1], Severin Gloor[1]

[1]Inselspital Bern, Universitätsklinik für Viszerale Chirurgie und Medizin, Bern, Schweiz

**Background:**
Electronic health record system (EHRS) transitions can disrupt surgical workflows and compromise patient safety. We evaluated the clinical and operational impact of transforming from multiple legacy systems to Epic within a high-volume surgical department.

**Material and methods:**
Investigator-initiated, retrospective, single-center, non-inferiority study conducted at Bern University Hospital comparing one year pre-implementation (March 1st, 2023 – March 1st, 2024) to one year post-implementation (March 2nd, 2024 – February 28th, 2025). Adult visceral surgery cases were included (n = 4,740). Primary endpoint was 30-day in-hospital mortality Secondary endpoints included postoperative complications, length of stay (LOS), logged working hours and intra-shift call volume. Analyses comprised descriptive comparisons, multivariable logistic regression models (adjusted for age, case-mix/day-mix, emergency status) and interrupted time-series (ITS) analyses of weekly outcomes.

**Results:**
30-day mortality remained unchanged (2.26% “pre-phase” vs. 2.26% “post-phase”, *p=0.702*) Adjusted ITS analyses showed no immediate level change but demonstrated a post-implementation slope decrease following a modest upward pre-trend . Overall complications increased (20.8% to 25.5%, p<0.001), driven by lower-grade events (notably grade II), while high-grade complications (≥ IIIa) remained stable. Mean LOS changed minimally (5.7 to 5.9 days, p=0.221); ITS suggested a transient increase around go-live followed by a downward post-trend. Staff workload analyses revealed small, heterogeneous shifts in logged hours (physicians: −0.81% per person, p=0.147; nurses: −2.00%, p<0.001) and a marked rise in call volumes across shifts (*p<0.001*) immediately after implementation.

**Conclusion:**
In a tertiary surgical setting, a large-scale EHRS transition was not associated with increased 30-day mortality, but coincided with a transient rise in postoperative complications and surge in intra-shift call volumes. Targeted support during early adoption, particularly for communication-intensive workflows, may help mitigate short-term safety and efficiency disruptions while maintaining patient survival outcomes.

DE GRUYTER BRILL

**Modified Johannesburg Technique Sets the Standard – Superior Stability in Chest Tube Fixation**
ID: E-DCK-200

**Chirurgie (auch interdisziplinär)/ Surgery (also interdisciplinary)**
**Krisen- und Kriegschirurgie / Crisis and War Surgery**

Johann Fricke[1], Ric Meißner[2], Stefan Schleifenbaum[2], Christian Kleber[1], Isabella Metelmann[3], Sebastian Krämer[3], Tobias Schöbel[1]

[1]Universitätsklinikum Leipzig, Klinik und Poliklinik für Orthopädie, Unfallchirurgie und Plastische Chirurgie, Leipzig, Deutschland, [2]ZESBO – Zentrum zur Erforschung der Stütz- und Bewegungsorgane, Leipzig, Deutschland, [3]Universitätsklinikum Leipzig, Klinik und Poliklinik für Viszeral-, Transplantations-, Thorax- und Gefäßchirurgie, Leipzig, Deutschland

**Background:**
Chest tube insertion is routine in thoracic and trauma surgery, yet tube dislocation remains a serious complication. While multiple fixation techniques exist, robust biomechanical comparisons are scarce, leaving technique selection largely to personal preference.

**Material and methods:**
This prospective, randomized experimental study evaluated 360 chest tubes of varying size (20 Ch, 24 Ch, 28 Ch) and material (PVC, silicone). Tubes were secured using 1–0 Prolene by two experienced thoracic surgeons with three suture techniques: modified Johannesburg, Roman sandal, and purse-string. Load-to-failure testing was conducted in four sessions at our biomechanical laboratory (ZESBO) using fresh porcine skin samples cut to size. The primary endpoint was the maximum force at failure. The secondary endpoint was the mechanism of failure.

**Results:**
The modified Johannesburg technique consistently demonstrated the highest pull-out strength across all tube sizes and materials. For 20 Ch tubes, mean maximum forces were 130 N (modified Johannesburg), 117 N (Roman sandal), and 94 N (purse-string) for silicone, and 195 N, 131 N, and 103 N for PVC, respectively. For 24 Ch tubes, silicone tubes showed mean forces of 178 N (modified Johannesburg), 131 N (Roman sandal), and 100 N (purse-string), while PVC tubes demonstrated 194 N, 137 N, and 100 N, respectively. Large-bore chest tubes (28 Ch) showed mean maximum forces of 170 N (modified Johannesburg), 135 N (Roman sandal), and 101 N (purse-string) for silicone, and 218 N, 143 N, and 102 N for PVC, respectively. Overall, the modified Johannesburg technique outperformed both Roman sandal and purse-string in every tested condition. The primary mechanism of failure was suture rupture (79%). Failure of the tube itself was most common for small (20 Ch) silicone tubes secured with the modified Johannesburg technique (85%).

**Conclusion:**
The modified Johannesburg technique provides superior mechanical stability in chest tube fixation. Given the clear advantage in pull-out resistance, its use has direct clinical relevance: reducing dislocation risk and thereby enhancing patient safety, especially in high-stress clinical and prehospital environments. These findings support the modified Johannesburg suture as the fixation technique of choice.

DE GRUYTER BRILL

**Turning on Green Light in the OR – a snapshot of surgical waste production and recycling potential**
ID: E-DCK-371

**Chirurgie (auch interdisziplinär)/ Surgery (also interdisciplinary)**
**Nachhaltigkeit in der Chirurgie / Sustainability in Surgery**

Niall Brindl[1 2], Jana Fellenberg[1], Colette Doerr-Harim[1], Christoph Michalski[1], Thilo Welsch[2], Julie Zangarini[3], Christopher Neuhaus[3], Andreas Brandl[1 2]

[1]Heidelberg University Hospital, General, Visceral and Transplantation Surgery, Heidelberg, Deutschland, [2]Krankenhaus Nordwest, General, Visceral and Tumour Surgery, Frankfurt, Deutschland, [3]Heidelberg University Hospital, Department of Anaesthesiology, Heidelberg, Deutschland

**Background:**
The medical sector is responsible for approximately 4–5% of global greenhouse gas emissions. In addition to the impact of buildings, energy use, patient transportation, and anaesthetic gases, medical waste and its processing add further to this environmental burden. The operating theatre, which nowadays relies heavily on single-use materials, is a particularly high producer of waste. This audit, conducted within the EU Horizon project Caring Nature, assessed the quantity and composition of waste generated by the surgical department at Heidelberg University Hospital to evaluate opportunities to reduce the ecological footprint of hospital care.

**Material and methods:**
A prospective waste audit was carried out over ten consecutive days in ten operating rooms, including seven general surgical theatres and one theatre each for vascular, heart, and urology specialties. Surgeries were classified by approach (open - minimally invasive), duration (minor, less than two hours - major, greater than two hours), urgency (elective - emergency), specialty, and subspecialty. Waste generated during each procedure, from anaesthetic induction through closure, was collected and sorted into glass, clean (non-contaminated plastics and paper, primarily packaging), and contaminated (materials that had contact with the patient). Sharps and infectious waste were excluded due to central collection. Descriptive statistics and group comparisons were calculated for waste volumes between approaches, specialties, and procedure types.

**Results:**
Acceptance of the audit was extraordinary within the whole team which took a lot of interest in the process and results. Compliance to the newly introduced waste separation was high throughout all professions.
A total of 151 operations were analysed. General surgery accounted for 42% of cases, with half represented by pancreatic resections. Urology contributed 22%, vascular 16%, heart 10%, transplant 3%, and other specialties 5%. On average, 0.51 kg (SD 0.42) of glass waste, 4.69 kg (SD 2.15) of contaminated waste, and 2.25 kg (SD 1.27) of clean waste were generated per surgery, yielding a mean total waste production of 7.45 kg (SD 2.78). Major surgeries ($n = 80$) produced significantly more waste than minor procedures ($n = 71$), with mean values of 8.58 kg (SD 2.82) compared to 6.24 kg (SD 1.61) respectively ($p < 0.001$), mainly due to greater amounts of both contaminated and clean waste. Minimally invasive operations (n = 24) generated slightly more waste than open procedures (n = 103), with mean amounts of 8.73 kg versus 7.55 kg, although this difference was not statistically significant. Cystoscopies in urology produced relatively little waste (5.42 kg, n = 24). No significant difference was observed between laparoscopic and robotic approaches (9.18 kg versus 9.13 kg). Emergency surgeries (n = 7) resulted in greater contaminated waste (6.27 kg vs. 4.60 kg) compared to elective procedures (n = 144), while clean waste was more abundant in elective cases (2.29 kg vs. 1.60 kg). Within general surgery, colorectal operations generated a mean of 7.23 kg total waste, hernia repairs 3.14 kg, liver surgery 9.00 kg, pancreatic resections 5.67 kg, and upper gastrointestinal procedures 7.55 kg; however, no significant difference was found among subspecialties (p = 0.187).

**Conclusion:**
Clean waste, consisting mainly of plastics and packaging, accounted for nearly one third of total waste and represents a promising target for recycling. This audit showed that segregation of medical waste is feasible and can be implemented in daily practice. It is one effective step toward more sustainable patient care.

DE GRUYTER BRILL

**Evidence Map of Appendicitis – a Living Systematic Review with Meta-Analyses**
ID: E-DCK-125

**Chirurgie (auch interdisziplinär)/ Surgery (also interdisciplinary)**
**Notaufnahme / Emergency Department**

Julian Harnoss[1], Dirk Kleindienst[2], Justyna Mohr[2], Katharina Maurer[2], Eva Kalkum[1], Pia Antony[2], Markus Müller[2], Pascal Probst[2]

[1]University Hospital Heidelberg, Study Center of the German Society of Surgery (SDGC), Dept. of General, Visceral and Transplantation Surgery, Heidelberg, Deutschland, [2]Cantonal Hospital Thurgau, Allgemein und Viszeralchirurgie, Münsterlingen und Frauenfeld, Schweiz

**Background:**
Appendicitis is one of the most common diseases of the gastrointestinal tract with a lifetime incidence of 7 to 9 %. Open and laparoscopic appendectomy are among the most performed emergency surgeries worldwide. Recently, more non-operative treatments have emerged. Given the large and growing body of evidence on appendicitis, it becomes difficult to maintain an overview. In order to assist in this endeavor, the aim of this project was to create a systematic and living Evidence Map of Appendicitis.

**Material and methods:**
PubMed, CENTRAL, Web of Science, and EMBASE were systematically and continuously searched for all published and ongoing randomized controlled trials (RCT) and systematic reviews (SR) dealing with the diagnosis and the treatment of appendicitis. Outcomes from every existing RCT were extracted and trial quality was assessed. RCTs and SRs on identical subjects were grouped to research topics. For specific treatment outcomes, meta-analyses were performed for all research topics with more than three RCTs.

**Results:**
Out of 13109 articles screened, 329 RCTs on 67354 patients and 324 SRs were included and grouped into 64 research topics with 22 living meta-analyses. Most RCTs were from Asia (47%), followed by Europe (29%) and North America (16%). Living meta-analyses were performed for 22 out of 64 research topics. For three out of 64 research topics (5%), there were no RCTs available.

**Conclusion:**
The Evidence Map of Appendicitis provides the ultimate go-to source for evidence on appendicitis. It is constantly updated and freely accessible via www.EVIglance.com and as a mobile phone app. Clinical decision-making and evidence-based patient information are supported by the primary data provided, as well as by living meta-analyses.

**Systematic review registration:** PROSPERO 2023 CRD42023482686

DE GRUYTER BRILL

**Free intra-abdominal air – a paradigm shift in surgery?**
ID: E-DCK-174

**Chirurgie (auch interdisziplinär)/ Surgery (also interdisciplinary)**
**Notaufnahme / Emergency Department**

Robert Brauer[1], Ekkehard Walle[2], Sebastian Steil[3]

[1]Marienkrankenhaus Cochem, Chirurgische Abteilung, Cochem, Deutschland, [2]Marienkrankenhaus Cochem, Gastroenterologische Abteilung, Cochem, Deutschland, [3]Marienkrankenhaus Cochem, Institut für Radiologie, Cochem, Deutschland

**Background:**
For decades, the radiological detection of free intra-abdominal air without prior intervention, implied immediate surgical evaluation of a hollow organ perforation. In a single-center observational study over a period of nine months, three patients with evidence of large amounts of free intra-abdominal air were successfully treated conservatively without surgical intervention. This raises the question of whether, with careful patient selection, a paradigm shift in the indication for laparoscopy/laparotomy is possible when free intra-abdominal air is detected.

**Material and methods:**
Two women (20 years and 41 years) presented as an emergency, and one man (78 years) presented as an incidental radiological finding, to the Marienkrankenhaus Cochem hospital within the last nine months. Abdominal CT scans revealed large amounts of free intra-abdominal air but no free fluid in all three patients. Both women initially presented with upper abdominal tenderness, while the man was completely asymptomatic. CT scans of both women revealed gastric wall thickening with CT morphological suspicion of a gastric ulcer. The man's CT scan was unremarkable except for sigmoid diverticulosis without wall thickening or signs of diverticulitis. All patients were admitted to the hospital and treated with intravenous antibiotics (Cefuroxime/Clont) and pantoprazole 2x40 mg intravenously and intravenous fluid therapy, and they were kept fasting. The 20-year-old patient underwent gastroscopy, and the gastric ulcer was debrided with clips. The 41-year-old patient refused any further intervention. The man underwent an ultrasound examination. His laboratory tests for inflammation were checked daily. Clinical visits were conducted twice daily for re-evaluation.

**Results:**
Free air was consistently detectable in all patients, including follow-up CT scans. All three patients had only mild clinical symptoms with pressure pain in the upper abdomen, without guarding. In addition to the moderate symptoms, the absence of free intra-abdominal fluid was also identical. In two patients, CT scans suggested a suspected perforated gastric ulcer.  In one patient, the ulcer was even endoscopically detected and monitored. In one patient, pneumatosis cystoides intestinalis was demonstrated sonographically (Fig. 4). CRP levels increased significantly in the patients with a perforated gastric ulcer, while leukocyte counts remained nearly normal, followed by gradual clinical improvement. All three patients discharged from the hospital without symptoms after five, seven, and nine days.

**Conclusion:**

With careful patient selection, under certain circumstances, even the detection of free intra-abdominal air, the paradigm of emergency laparoscopy/laparotomy can be deviated from. Due to its muscular permeability, the gastric wall can, in some cases, seal itself off and act merely as a valve. The following were identical in all cases:- the absence of leukocytosis, a sign of the absence of peritonitis,- the only mild and steadily regressing clinical symptoms, and- no evidence of free intra-abdominal fluid on CT.Requirement for a conservative approach are multiple daily clinical monitoring with a willingness to adapt the approach, as well as close interdisciplinary collaboration between gastroenterologists, visceral surgeons, and radiologists.

DE GRUYTER BRILL

**Who Cares for the Critically Ill Surgical Patient: Expectations and Reality**
ID: E-DCK-320

**Chirurgie (auch interdisziplinär)/ Surgery (also interdisciplinary)**
**Perioperative Medizin / Perioperative Medicine**

Tobias Schulze[1], Stephan Kersting[1]

[1]Universitätsmedizin Greifswald, Klinik für Allgemeine Chirurgie, Viszeral-, Thorax- und Gefäßchirurgie, Greifswald, Deutschland

**Background:**
Since the late 1990s, specialty-based intensive care units have increasingly been converted into larger interdisciplinary intensive care units. While in anesthesiology and internal medicine, the growing importance of intensive care medicine—driven by an increasingly older and multimorbid patient population as well as the rising complexity of medical treatments—was acknowledged, and intensive care developed into an independent core discipline, perioperative intensive care of surgical patients has remained only one of many routine clinical tasks in surgery. As a result, leadership roles in intensive care were increasingly taken over by anesthesiologists. This development was extensively discussed at the 2011 Congress of the German Society of Surgery, leading to recommendations aimed at safeguarding the continued existence of surgical intensive care as an integral part of surgical practice [1,2]. In 2025, Wildenauer and Hamsen investigated the structure of surgical intensive care medicine in Germany and demonstrated that surgical ICUs were largely limited to university hospitals, while their presence in hospitals of lower care levels was the exception [3]. The present study provides a descriptive analysis of the current structure of training in intensive care medicine (Zusatzbezeichnung “Intensivmedizin”) and of perioperative intensive care in Germany.

**Material and methods:**
All German State Medical Associations (Landesärztekammern) were asked to provide quantitative data on postgraduate training in intensive care medicine (Zusatzbezeichnung “Intensivmedizin”). Data on the number of physicians authorized to provide such training were obtained from the official registries of the German State Medical Associations (Landesärztekammern). Chief surgeons in hospitals with at least 300 beds were personally contacted by email and invited to complete an online questionnaire hosted on the platform SoSci Survey (SoSci Survey GmbH, Munich). Data were collected on structural characteristics of perioperative intensive care for surgical patients and on subjective views of senior surgeons regarding the role of surgeons in perioperative intensive care.

**Results:**

Of the 17 German State Medical Associations, 10 (58.8%) responded, and 6 (35.2%) were able to provide data with the desired level of detail. Among the total of 556,200 physicians registered in 2022, 173,057 (31.1%) were members of these 6 associations. In 2022, a total of 29 surgeons obtained the additional qualification “Intensive Care Medicine,” compared to 174 anesthesiologists and 82 internists. Nationwide, 1,700 physicians were authorized to provide postgraduate training in intensive care medicine, 4.5% of them with a background in surgical specialties (excluding neurosurgery). The majority of trainers were anesthesiologists and internists (47.8% and 30.6%, respectively).
Of the 472 hospitals with more than 300 beds that were contacted, 41 senior physicians (8.6%) responded; 37 questionnaires were fully completed and included in the analysis. In 2023, perioperative intensive care was provided in surgical-led ICUs in only 4 of the surveyed hospitals (10.8%).

**Conclusion:**

Under current clinical conditions, the demand expressed in 2011 by several leading surgeons to systematically involve surgeons in the leadership of intensive care units caring for surgical patients cannot be realized. The number of surgeons with formal training in intensive care medicine appears insufficient to meet the structural requirements defined by DIVI and DIMDI. If perioperative care of surgical patients is to remain an integral part of surgical practice, urgent measures are needed to expand training capacity and implement structural incentives to motivate surgeons to pursue careers in intensive care medicine.

Biblography:

1. Sarikouch S, Haverich A. Der chirurgische Einfluss auf die Intensivmedizin muss sich vergrößern. Zentralbl Chir 2011; 136(2): 101.
2. Muhl E. Chirurgische Intensivmedizin – Stellenwert für chirurgische Weiterbildung und Versorgungsstruktur heute und in der Zukunft. Zentralbl Chir 2011; 136(2): 102-105.
3. Wildenauer R, Hamsen U. [Development of surgical intensive care medicine and status quo in Germany]. Chirurgie 2025; 2025(19): 025-02320.

**Intraoperative vital sign variability as early warning signals of postoperative infection**
ID: E-DCK-372

**Chirurgie (auch interdisziplinär)/ Surgery (also interdisciplinary)**
**Perioperative Medizin / Perioperative Medicine**

Tobias Blatter[1], Yves Wintsch[1], Karen Triep[2], Olga Endrich[2 3], Hugo Armando Guillen-Ramirez[1], Guido Beldi[1]

[1]Department of Visceral Surgery and Medicine, Inselspital, Bern University Hospital, University of Bern, Bern, Switzerland, Bern, Schweiz, [2]Medical Directorate, Medizincontrolling, Inselspital, University Hospital Bern, Insel Gruppe, Bern, Schweiz, [3]Department of Clinical Chemistry, Inselspital, Bern University Hospital, University of Bern, Bern, Schweiz

**Background:**
Postoperative bacterial infections are among the most frequent surgical complications, leading to prolonged hospital stay, higher costs, and increased morbidity. Identifying at-risk patients immediately after surgery remains a major challenge. While preoperative risk models exist, intraoperative physiological dynamics have not been fully leveraged for infection prediction.

**Material and methods:**
We conducted a retrospective cohort study of 16,766 surgical cases at a tertiary hospital (2014–2022). Bacterial infection was defined by curated ICD-10 codes. Preoperative variables included age, sex, ASA score, and comorbidities. Intraoperative time-series data were extracted for arterial blood pressure, heart rate, peripheral oxygen saturation ($SpO_2$), nasopharyngeal temperature, and end-tidal $CO_2$. Statistical and dynamic features were derived, including higher order metrics (skewness, kurtosis, entropy, inter-event intervals, and trends). Postoperative laboratory markers at POD2 were incorporated using imputation. Machine learning models (XGBoost, Random Forest, logistic regression) were trained to predict infection risk at three time points: before surgery, at the end of surgery, and POD2. Model interpretability was assessed using SHAP values.

**Results:**
Postoperative infections occurred in 5.9% of surgeries. Predictive performance improved across time points: AUROC 0.77 (preoperative), 0.87 (end of surgery), and 0.91 (POD2 with labs). Intraoperative higher order features strongly contributed to prediction, including median $SpO_2$ (protective), maximum intraoperative temperature (risk), blood pressure kurtosis, and heart rate inter-event intervals. Specificity remained high across models (0.96–0.99), while sensitivity improved from 0.26 preoperatively to 0.40 at POD2. Calibration was good across procedure clusters, with minor underestimation of infection risk in low-volume clusters.

**Conclusion:**
Postoperative infection risk can be reliably predicted as early as the end of surgery using intraoperative vital sign dynamics. This approach enables earlier risk stratification, even before routine labs are available, and provides interpretable, clinically actionable signals. Leveraging intraoperative monitoring data could support real-time perioperative decision-making and targeted postoperative surveillance.

DE GRUYTER BRILL

**AI-derived perioperative timelines for disentangling mortality and infection risk stratification**
ID: E-DCK-383

**Chirurgie (auch interdisziplinär)/ Surgery (also interdisciplinary)**
**Perioperative Medizin / Perioperative Medicine**

Katharina Lucas[1], Yves Wintsch[1], Tobias Blatter[1], Karen Triep[2], Olga Endrich[2 3], Hugo Armando Guillen-Ramirez[1], Guido Beldi[1]

[1]Department of Visceral Surgery and Medicine, Inselspital, Bern University Hospital, University of Bern, Bern, Switzerland, Bern, Schweiz, [2]Medical Directorate, Medizincontrolling, Inselspital, University Hospital Bern, Insel Gruppe, Bern, Schweiz, [3]Department of Clinical Chemistry, Inselspital, Bern University Hospital, University of Bern, Bern, Schweiz

**Background:**
Postoperative mortality and bacterial infection are frequent yet mechanistically distinct complications. Clinical practice often treats them separately, yet perioperative data suggest overlapping signals that evolve over time. We aimed to evaluate whether electronic health record (EHR) data can identify outcome-specific risk windows across surgical specialties.

**Material and methods:**
We retrospectively modelled 30-day mortality and postoperative infection using 33,775 perioperative EHR records across seven procedure groups (thoracic, cardiac, vascular, abdominal wall, gastrointestinal, hepatobiliary, urologic). Predictors included baseline/frailty and operative context (e.g., ASA, emergency or night surgery, duration) and laboratory values from POD 0–7. For each outcome, we trained time-resolved models and examined day-by-day predictor influence and specialty-specific patterns. Performance was assessed by AUROC, sensitivity, specificity, and F1-score across daily windows and procedure clusters.

**Results:**
Mortality models achieved high discrimination (AUROC up to 0.90 across clusters), driven by baseline frailty and early haemoglobin/renal dynamics. However, recall was low (<0.4), reflecting the rarity of events, while specificity remained >0.95. In contrast, infection models showed moderate discrimination (AUROC 0.75–0.82) but better recall (0.6–0.7), particularly after POD 2 when CRP, platelet rebound, and renal trends emerged as key predictors. Specificity ranged 0.75–0.85. This contrast suggests an early frailty window (POD 0–2) for mortality and a later trajectory window (POD 3–7) for infection.

**Conclusion:**
Time-resolved EHR models reveal distinct temporal signatures of mortality and infection: early signals suited for triage and haemostasis; later signals reflecting evolving physiological trajectories. While further calibration and validation are ongoing, these findings highlight complementary windows for outcome-specific monitoring and prevention strategies.

DE GRUYTER BRILL

**Improving Communication in the OR: A Pre-Post Analysis after Introduction of Name Tags**
ID: E-DCK-430

**Chirurgie (auch interdisziplinär)/ Surgery (also interdisciplinary)**
**Perioperative Medizin / Perioperative Medicine**

Alexander Bungert[1], Jan Philipp Ramspott[1], Carsten Szardenings[2], Michael Klatthaar[3], Tim Güß[3 4], Alina Knipping[1], Benjamin Struecker[1], Andreas Pascher[1], Jens Peter Hölzen[1]

[1]Klinik für Allgemein-, Viszeral- und Transplantationchirurige, Universitätsklinikum Münster, Münster, Deutschland, [2]Institute of Biostatistics and Clinical Research, University of Muenster, Münster, Deutschland, [3]UKM Trainingszentrum, Universitätsklinikum Münster, Münster, Deutschland, [4]Klinik für Anästhesiologie, operative Intensivmedizin und Schmerztherapie, Universitätsklinikum Münster, Münster, Deutschland

**Background:**
Effective communication is crucial in the operating room (OR), yet miscommunication remains a major cause of adverse events. Although the World Health Organization (WHO) Surgical Safety Checklist (SSC) was introduced to manage critical steps during surgery, its implementation alone has not fully addressed interpersonal recognition and familiarity. Proper communication in high-risk areas like the OR becomes more important as robotically assisted surgeries are increasingly being carried out. In this scenario face-to-face communication is limited representing a challenge in case of medical emergencies. This study investigates whether color-coded name tags can improve team familiarity and perceived team communication.

**Material and methods:**
A pre-post survey study was conducted at a tertiary university hospital in Germany. The intervention consisted of the implementation of color-coded name tags by professional group. The pre-intervention questionnaire (19 items including free text) was distributed to more than 600 employees of the OR staff (most of surgical disciplines, anaesthesia, cleaning and service staff) in October 2024. The post-intervention questionnaire (38 items) includes validated questions to patient safety and will be sent in October 2025. No financial incentives are intended.

**Results:**
Preliminary results from the pre-intervention questionnaire revealed strong adherence with the SSC but persistent deficits in communication. Mutual familiarity levels are not as high as expected in a high-risk medical area. Name tagging has been regarded as a potential tool to expand familiarity levels and enhance psychological safety.

**Conclusion:**
Communication is a prerequisite for good surgical results. However, miscommunication in the operating theatre has the potential to harm patients long-term. In our study we could show that name tagging in the operating theatre is a simple low-cost intervention that has a significant impact on perceived communication and could improve patients' safety.

DE GRUYTER BRILL

**Perioperative aspects causing parental distress in pediatric surgical patients**
ID: E-DCK-484

**Chirurgie (auch interdisziplinär)/ Surgery (also interdisciplinary)**
**Perioperative Medizin / Perioperative Medicine**

Christina Campbell[1], Felicitas Eckoldt[1]

[1]Universitätsklinikum Jena, Klinik für Kinderchirurgie, Jena, Deutschland

**Background:**
It has been well established that surgery during childhood can cause severe distress to children and their caregivers. Parental distress hereby has been shown to correlate positively with not only distress perioperatively but also with post-traumatic symptoms of children months after surgery and discharge from the hospital. Although many children with post-medical trauma stress symptoms after undergoing surgery experience a spontaneous resolve of these, a relevant number of patients may develop chronic PTSD symptoms. This implies long-term sequelae which will necessitate further treatment or make any further necessary and especially any invasive treatment more difficult and costly in terms of resources. Recurrent surgical procedures seem to be a risk factor for the development of PMTS (post-medical trauma stress) symptoms just as parental anxiety and many other factors.

**Material and methods:**
In order to assess factors contributing to parental distress and thereby increasing the risk for our patient population being treated at the Pediatric Surgery Department at the University Hospital Jena to experience PMTS symptoms we conduct a structured written interview with parents of children of the ages 0-8years undergoing surgery.
In order to find relevant points in question, nursing staff as well as physicians and a psychologist handling postoperative cases on our pediatric surgical ward were questioned in open questions. Recurring answers were pooled, structured and further questions pertaining to risk factors evident from literature were added. The interview is conducted anonymously within 48 hours of the child's surgery with the parent staying with the child. A screening tool was used to assess underlying psychosocial risk factors and parental distress symptoms. Nature (elective vs emergent) of intervention, subjective impression of being informed well, having spoken a member of the operating team pre-/postoperatively, recurrent surgery, whether or not communicated estimated length of surgery were consistent with reality, gender of parent as well as patient and socioeconomic status were assessed. Written information was handed out and consent obtained together with the interview form.

**Results:**
While surgery of a child remains a stressful event for parents, reducing parental distress seems to be possible through thorough and fitting communication. Risk of increased parental distress is composed of indivual risk factors; assessing these should be included during preoperative planning and decision-making. Communication with caretakers of children especially in surgical settings should include explaing the possible effects of parental distress on their children.

**Conclusion:**
Personnel which are trained and well-versed in the surgical care of the pediatric population should be the standard in modern pediatric surgical settings from our perspective and supported by the results of the interview. Further research is needed to tailor communication in these settings to individual parent-child risk components.

DE GRUYTER BRILL

**Deep Learning Applications for preoperative Head and Neck Tumor Staging: A Systematic Review**
ID: E-DCK-115

**Chirurgie (auch interdisziplinär)/ Surgery (also interdisciplinary)**
**Robotik und KI-gestützte Verfahren / Robotics and AI-Assisted Procedures**

Jannik Ketschau[1], Nils Krautkremer[1], Alex Grabenhorst[1], Hannes Singer[1], Helena Kram[1], Maximilian Miragall[1], Klaus-Dietrich Wolff[1], Lucas Ritschl[1]

[1]Technische Universität München, TUM Klinikum Rechts der Isar, Mund-, Kiefer-, und Gesichtschirurgie, München, Deutschland

**Background:**
Accurate preoperative classification of cervical lymph nodes (LNs) and primary tumor invasion is essential for staging and treatment planning in head and neck cancer (HNC). Conventional imaging suffers from interobserver variability and modest sensitivity for lymph node metastasis, extranodal extension (ENE) or local tumor invasion. Misclassification affects surgical decisions and radiotherapy planning. Deep learning (DL) methods have emerged as promising tools to improve diagnostic accuracy and reduce variability. However, the literature remains heterogeneous with respect to imaging modalities, annotation strategies, model architectures, and validation practices. While some studies show near-human or superior performance, others highlight methodological pitfalls. This review compiles current DL research for preoperative LN and tumor invasion assessment in HNC, focusing on methodological patterns, performance, and translational potential.

**Material and methods:**
This review followed PRISMA guidelines and included peer-reviewed studies published between 2018 and 2025 that developed and validated DL models for LN classification or local tumor invasion in HNC. Eligible tasks included LN metastasis prediction and detection, ENE detection, and assessment of tumor infiltration into adjacent structures. Exclusion criteria were non-English publications, non-DL methods, segmentation-only tasks without classification, reviews, or studies lacking clinical endpoints. A structured Pubmed search was performed in three steps: an initial query on DL model training in head and neck pathology (124 records, 67 retained), an expanded search with segmentation/classification and cancer-related terms (861 records, 187 retained), and a final query targeting staging and diagnostic modeling (302 records, 11 retained). After re-evaluation, 23 studies fulfilled inclusion criteria. Data extracted included study design, population, imaging modality, annotation type, dataset size and split, preprocessing, model architecture, training strategy, validation, and performance metrics. External validation, reader comparison, and bias assessment were specifically noted.

**Results:**
Twenty-three studies were included: 18 on LN classification and 5 on tumor invasion. Most were retrospective single-center studies (n=21), with only two prospective or multicenter cohorts. Sample sizes ranged from 39 to 1,236 patients, with some LN studies analyzing thousands of segmented nodes. CT was the dominant modality (n=17), followed by MRI (n=3) and PET/CT (n=2); one study used bone scintigraphy with MRI reference. Annotation strategies varied substantially. Several LN studies employed slice-level labeling, often aggregating predictions across multiple slices for a patient-level decision. Others relied on LN segmentations derived from contrast-enhanced CT or MRI. Tumor invasion studies used pathology-confirmed labels or consensus radiologist readings as reference standards. While some studies provided carefully delineated masks of invaded regions, others used broader region-of-interest annotations or relied on post-hoc labeling of consecutive positive slices, a practice associated with potential bias. CNN-based models predominated with U-Net components for segmentation, DualNet 3D CNNs for ENE, Mask R-CNN for LN detection, and frequent use of ResNet backbones for transfer learning. Fusion models incorporating radiomics or clinical data were also reported. Dataset sizes varied widely, from <100 patients to >20,000 CT slices. Seventeen studies performed external validation, and six included reader comparisons. Performance was consistently high with LN classification achieving AUCs of 0.82–0.98, ENE detection up to 0.91, and tumor invasion models 0.80–0.94. Reader studies showed AI support often improved sensitivity and accuracy, in some cases to near-perfect levels. Bias assessment revealed recurrent risks, including small external validation sets, single-center design, MRI heterogeneity, and occasional thresholding on test sets. Reproducibility was limited, with few studies sharing code or data. Clinical relevance was addressed explicitly in several studies. LN classification systems were proposed as decision support tools to refine preoperative staging and radiotherapy planning. ENE detection models demonstrated utility as triage aids, outperforming radiologists in early validation. Tumor invasion models were positioned as support for detecting subtle infiltration, with reader studies confirming that AI support improved diagnostic sensitivity and confidence.

**Conclusion:**
Deep learning models show strong promise for preoperative LN and tumor invasion classification in HNC, often matching or exceeding radiologists. Clinical utility is supported by reader studies, particularly for ENE detection and subtle invasion. However, methodological limitations, small, retrospective cohorts, inconsistent annotation standards, limited robustness testing, and insufficient reproducibility, remain significant barriers. Future work should emphasize prospective multicenter trials, standardized references, external validation, and integration of calibration and uncertainty estimation. With these refinements, DL systems could become reliable adjuncts to radiologists, supporting more precise and consistent decision-making in HNC staging and treatment planning.

DE GRUYTER BRILL

**The Neuro-Symbolic Anatomy Engine: A Novel Architecture for Universal Anatomical Modelling**
ID: E-DCK-164

**Chirurgie (auch interdisziplinär)/ Surgery (also interdisciplinary)**
**Robotik und KI-gestützte Verfahren / Robotics and AI-Assisted Procedures**

Stefan Rolf Benz[1 2], Anne Benz[2], André Mihaljevic[3], Jule Reitz[2], Paul Benz[2]

[1]Kliniken Böblingen, Klinikverbund-Suedwest, Allgemein-, Viszeral-; Thorax- und Kinderchirurgie, Böblingen, Deutschland, [2]Firma Simultare, Nagold, Deutschland, [3]Universitätsklinik Tübingen, Allgemein-, Viszeral und Transplantationschirurgie, Tübingen, Deutschland

**Background:**
The current landscape of surgical education is facing a critical juncture, characterized by a growing disparity between training capacity and the demands of modern surgical practice. This gap, which we term the "surgical training trap," has significant implications for patient safety, healthcare costs, and the advancement of surgical innovation. To address these challenges, we propose a paradigm shift in surgical simulation through the development of a Neuro-Symbolic Anatomy Engine.
The crisis in surgical education is multifaceted, stemming from economic constraints, workforce evolution, and the increasing complexity of surgical techniques. Traditional training methods are struggling to keep pace with rapid surgical innovations, resulting in a narrowing of the surgical competence spectrum. This is particularly evident in the difficulty of advancing surgeons to competently perform complex procedures, such as robotic CME colectomy or pancreatectomy, despite their ability to master simpler operations.
The consequences of this training gap are substantial, albeit often concealed due to the delayed manifestation of adverse outcomes in a surgeon's career. Many surgeons complete formal training and obtain board certification without achieving independence in high-complexity operations. This disconnect extends beyond residency, as surgeons frequently expand their operative repertoire through limited means such as reading and watching videos, leading to a significant proportion of complex operations being performed on some form of learning curve.
These ongoing learning curves pose risks to patient safety, with increased complications and mortality rates associated with inexperience (1, 2). Moreover, the financial impact is considerable, as complications lead to longer hospital stays, additional interventions, and inflated healthcare costs (3).

**Material and methods:**
To address these challenges, we propose the Neuro-Symbolic Anatomy Engine, a hybrid AI architecture that generates a comprehensive, high-resolution representation of human anatomy. This system integrates neural network processing of text and images with a symbolic knowledge-based framework, enabling the simulation of any surgical procedure within a complete anatomical workspace.
The proposed engine comprises several key components:

1. A knowledge representation layer maintaining a comprehensive model of human anatomy, including topographical relationships, physiological properties, and cause-effect chains.
2. A symbolic reasoning engine for logical inference over anatomical relationships, ensuring consistency with established medical knowledge.
3. Neural perception modules for multimodal input processing, including natural language processing, image analysis, and sensor data interpretation.

4. An integration interface facilitating bidirectional communication between symbolic and neural components.

This architecture addresses limitations of current simulation systems based on frameworks like VTK (Visualization Toolkit) or Gmsh, which lack the ability to leverage existing anatomical knowledge or reason about anatomical relationships logically. The Neuro-Symbolic Anatomy Engine aims to create a deep understanding of anatomy, allowing for topographical predictions even when structures are not directly visible.

**Results:**
A proof-of-concept model demonstrates the feasibility of this approach, converting anatomical prose into a two-dimensional knowledge graph visualizing spatial and functional relationships. While this initial model has limitations, it serves as a foundation for future development.
The proposed system offers several potential advantages:

1. Interpretability: The explicit symbolic representation of anatomical relationships allows for systematic identification and correction of errors, which is crucial in medical applications (12).
2. Data Efficiency: Neuro-symbolic approaches have shown promise in structured domains, potentially enabling accurate predictions even with limited training data (9).
3. Scalability: The modular architecture theoretically supports incremental expansion and distributed processing, although practical scalability challenges remain.

However, significant challenges must be addressed, including the computational complexity of real-time anatomical reasoning, knowledge base maintenance, and the need for extensive clinical validation.

**Conclusion:**
The Neuro-Symbolic Anatomy Engine represents a novel approach to surgical simulation that could deeply transform surgical education and innovation. By creating a manipulable and dynamic AI model of human anatomy and physiology, this system has the potential to greatly elevate surgical excellence and safety, while accelerating surgical education to meet contemporary demands. As AI technology rapidly evolves, the time is ripe to embark on this ambitious endeavour.

DE GRUYTER BRILL

**Using AI and Computational Simulations for Liver Tumor Assessment and Surgical Decisions**
ID: E-DCK-260

**Chirurgie (auch interdisziplinär)/ Surgery (also interdisciplinary)**
**Robotik und KI-gestützte Verfahren / Robotics and AI-Assisted Procedures**

Anton Schnurpel[1], Matthias König[2], Mojtaba Nayyeri[3], Sandra Nickel[4], Luis Mandl[3], Steffen Staab[5], Tim Ricken[5], Hans-Michael Tautenhahn[4]

[1]Universitätsklinikum Leipzig, Experimentelle Hepatobiliäre Chirurgie, Leipzig, Deutschland, [2]Humboldt-Universität zu Berlin, Institut für Theoretische Biologie, Berlin, Deutschland, [3]Universität Stuttgart, Institut für Parallele und Verteilte Systeme, Stuttgart, Deutschland, [4]Universitätsklinikum Leipzig, Klinik und Poliklinik für Viszeral-, Transplantations-, Thorax- und Gefäßchirurgie, Leipzig, Deutschland, [5]Universität Stuttgart, Institut für Statik und Dynamik der Luft- und Raumfahrtkonstruktionen, Stuttgart, Deutschland

**Background:**
With over 500,000 new cases annually, cancer remains a major challenge for the German healthcare system. Liver tumors represent the fifth most common cancer worldwide and the second leading cause of cancer-related death. To provide patients with hepatocellular carcinoma (HCC) and other liver malignancies with the most effective therapy, treatment strategies are regularly discussed in interdisciplinary tumor boards. Although various therapeutic options for HCC have been developed over the last decade, the choice of a patient-specific therapy remains extremely challenging, as it depends on multiple factors such as tumor type, age, sex, comorbidities, and the patient's overall condition. Current decision-making often relies on static classification systems such as the BCLC score, which provides only a rigid framework and cannot reflect the complexity of highly individual patient cases or the specific clinical context on-site. At the same time, large amounts of data generated in everyday clinical practice remain unused, although they could offer new insights by highlighting correlations between patient characteristics, lifestyle, disease progression, and outcomes. Even highly experienced clinicians face difficulties in capturing and interpreting nonlinear interdependencies between these factors and their interplay with therapeutic options. While tumor boards aim to integrate different perspectives, they often cannot fully cover the required multidisciplinarity, and highly specific cases may not be addressed by existing guidelines. In addition, consensus-building in tumor boards is frequently delayed by the multitude of specialist opinions, which can be particularly problematic in time-critical decision phases. Consequently, the management of liver tumors requires a multidimensional, multidisciplinary, and dynamic decision support strategy that leverages patient-specific data and supports clinicians in making timely and individualized therapy decisions.

**Material and methods:**
To enable objective, transparent, and individualized treatment decisions, we are developing ATLAS (AI and Simulation for Tumour Liver Assessment), a Clinical Decision Support System (CDSS). ATLAS integrates machine learning with patient-specific computational liver models and applies clustering approaches within a dynamic, time-resolved knowledge graph that captures structured clinical data. For AI training and model simulation, a multicentric database comprising structured tabular data as well as 2D and 3D imaging data is employed. The ATLAS-AI combines these heterogeneous inputs to generate traceable treatment recommendations. In addition, an embedded large language model (LLM) improves accessibility by enabling intuitive interaction with the data and context-aware reasoning, even for users without technical expertise.

**Results:**
A functional demonstrator of the CDSS has been tested in simulated tumor boards. It enables the visualization and prediction of patient outcomes under various therapeutic scenarios, including different surgical approaches such as liver transplantation. This functionality supports refined risk stratification and treatment prioritization, extending beyond conventional BCLC rules and rigid, non-personalized guidelines. By relying on a standardized clinical database, the system establishes a robust and temporally consistent foundation for outcome analysis. The seamless integration of a liver tumor knowledge graph enables patient stratification by linking individual cases to others with comparable disease trajectories. This not only facilitates overall comparisons but also provides valuable insights by contextualizing a patient’s condition in relation to clinically similar cases.

**Conclusion:**
The development of ATLAS demonstrates the feasibility and potential of AI-driven decision support in the management of liver tumors. By integrating machine learning, computational liver modeling, and a dynamic knowledge graph, the system goes beyond rigid classification schemes and enables individualized, data-driven treatment recommendations. The functional demonstrator tested in simulated tumor boards highlights the capacity of ATLAS to visualize therapeutic options, predict patient outcomes, and support refined risk stratification. In doing so, it addresses critical challenges of current tumor board practice, including delayed consensus-building, and underutilization of routine clinical data. Future work will focus on validating the system with real-world patient data and ensuring seamless integration into clinical workflows, with the ultimate goal of enhancing decision-making, improving patient outcomes, and advancing precision oncology in liver cancer care.

DE GRUYTER BRILL

**Readability Analysis of German Patient Education Materials (PEM) on Cleft Lip and Palate**
ID: E-DCK-4

**Chirurgie (auch interdisziplinär)/ Surgery (also interdisciplinary)**
**Robotik und KI-gestützte Verfahren / Robotics and AI-Assisted Procedures**

Juliane Kröplin[1], Michael Prestele[2], Bernhard Frerich[1], Sabine Drossard[3]

[1]University Medical Centre Rostock, Rostock, Deutschland, [2]Ludwig-Maximilians-University Munich, Munich, Deutschland, [3]University Hospital Würzburg, Würzburg, Deutschland

**Background:**
Cleft lip and palate treatment requires complex, long-term care, making health literacy especially important for new parents.  This study aims to evaluate the readability of German PEM on cleft lip and palate and to test whether AI can improve readability without compromising medical accuracy.

**Material and methods:**
University hospitals in Germany were identified via the German Society of Oral and Maxillofacial Surgery website. The readability of existing PEM on the treatment for cleft lip and palate was analysed using two validated readability indices for German Language „Lesbarkeitsindex“ (LIX) and "Flesch-Reading-Ease Score” (Flesch). The texts were then adapted using ChatGPT. Following verification for medical accuracy, the mean values of the LIX and Flesh indices for the rephrased texts were compared to those of the original texts.

**Results:**
N=20 university hospitals were analysed. N=11 provided further information on the treatment procedure for cleft lip and palate. The average LIX index of the existing PEM was 57.3 (medium difficult); the average Flesch index was 28 (very difficult). The readability of the analysed materials could be significantly simplified for both indices ($p<.001$).

**Conclusion:**
Existing German PEM on cleft lip and palate are mainly classified as “difficult” to read. AI has the potential to assist in simplifying PEM, improving their readability. PEM in plain language could be a useful addition to the information currently available on the treatment of cleft lip and palate. This might help to improve the health literacy of new parents of all educational levels.

DE GRUYTER BRILL

### AI transferability between different hospitals via phase recognition in laparoscopic cholecystectomy

ID: E-DCK-98

**Chirurgie (auch interdisziplinär)/ Surgery (also interdisciplinary)**
**Robotik und KI-gestützte Verfahren / Robotics and AI-Assisted Procedures**

Sebastian Lünse[1], Lasse Renz-Kiefel[2], René Mantke[1], Peter Eisert[2], Anna Hilsmann[2], Eric Wisotzky[2]

[1]University Hospital Brandenburg, Brandenburg Medical School, Department of Surgery, Brandenburg, Deutschland, [2]Fraunhofer Heinrich-Hertz-Institute, Vision & Imaging Technologies, Berlin, Deutschland

**Background:**

The growing complexity of surgical procedures, driven by digital support tools in the operating room, underscores the importance of analyzing surgical workflows to improve outcomes. Central to this analysis is the recognition of distinct surgical phases, which enables a deeper understanding of procedural dynamics and supports training, assistance, and optimization efforts. In laparoscopic surgery, particularly laparoscopic cholecystectomy (LC), phase recognition is crucial due to the technical challenges and variability in surgical workflows. Machine learning (ML) models have been widely used for surgical phase recognition, but their effectiveness is limited by the availability of annotated data and the diversity of surgical practices across clinics. This study addresses the challenges of model generalization and transferability by investigating various training and fine-tuning strategies on both public and proprietary datasets, aiming to improve the adaptability of ML models to unseen clinical environments and enhance their practical applicability in real-world surgical settings.

**Material and methods:**

We used three datasets—Cholec80, HeiChole, and our own Medical School Brandenburg (MHB) dataset—containing a total of 125 laparoscopic cholecystectomy videos to train machine learning models for surgical phase recognition. The datasets vary in recording conditions and video characteristics, with Cholec80 being the most homogeneous and MHB the most heterogeneous. A two-stage deep learning architecture (TeCNO), based on ResNet50 and multi-stage temporal convolutional networks (MS-TCN), was employed for feature extraction and recognition of seven defined phases (P1 to P7). We conducted multiple experiments to evaluate model transferability across different datasets using a nested 5-fold cross-validation approach to ensure robust and unbiased results. The experiments explored various training strategies, including the integration of public and own data, and fine-tuning on new clinical environments to simulate real-world applications. The evaluation was carried out exclusively on the MHB dataset for all experiments.

**Results:**

The conducted experiments investigated the transferability and performance of a surgical phase recognition model (TeCNO) across different datasets. When trained exclusively on the MHB dataset (Exp. A), the model achieved an average accuracy of 79.66%, which was more than 10% lower than models trained and tested on the homogeneous Cholec80 dataset. Using public datasets alone (Exps. B and C) resulted in decreased performance and significant confusion between phases, especially between P2 and P4. Combining both public datasets (Exp. D) improved accuracy by 2–6%, and further increasing the training set (Exp. E)

showed incremental improvements. Adding some MHB data (Exp. F) yielded the highest accuracy of 84.30%, significantly outperforming earlier experiments. Fine-tuning with MHB data (Exp. G) resulted in slightly lower performance but still demonstrated substantial improvements over baseline models.

**Conclusion:**
This study investigates the transferability of machine learning models for surgical phase recognition across hospitals using the MHB dataset and public datasets (Cholec80, HeiChole). The best model achieved 84% accuracy on MHB data, showing that combining diverse datasets improves performance, though it remains below the reference benchmark trained solely on public data. Fine-tuning with local data boosts performance but does not outperform re-training from scratch, highlighting the need for clinic-specific data. Errors in phase prediction stem from inconsistent phase definitions and varying surgical workflows across clinics, suggesting that task-based recognition might enhance transferability. The study underscores the necessity of diverse, multi-center datasets to build robust and transferable ML models for surgical applications.

DE GRUYTER BRILL

**Feasibility and Limitations of Robotic-Assisted Visceral Pediatric Surgery**
ID: E-DCK-99

**Chirurgie (auch interdisziplinär)/ Surgery (also interdisciplinary)**
**Robotik und KI-gestützte Verfahren / Robotics and AI-Assisted Procedures**

Karolin-Marie Jungjohann[1 2], Maximilian Bernhardt[1 2], Gennadii Ivanov[1 2], Katja Reischig[1 2], Robert Bergholz[1 2], Andreas Meinzer[1 2]

[1]University Hospital of Schleswig-Holstein, Campus Kiel, Department for General, Visceral, Thoracic, Transplantation and Pediatric Surgery, Arnold-Heller-Straße 3, 24105, Kiel, Deutschland, [2]Kurt-Semm-Centre for endoscopic, laparoscopic and robotic minimally invasive Surgery Kiel, University Hospital of Schleswig-Holstein, Campus Kiel, Arnold-Heller-Straße 3, 24105, Kiel, Deutschland

**Background:**
Robotic-assisted surgery is well established in adults but rarely applied in children due to body size limitations and the lack of pediatric-specific instruments. This study evaluates feasibility, complications and limitations of robotic visceral surgery in a pediatric cohort.

**Material and methods:**
A retrospective and ongoing prospective analysis is performed of all pediatric patients undergoing robotic-assisted procedures at the Department of Pediatric Surgery, University Hospital Schleswig-Holstein, Kiel, between 2014 and 2025. The collected and analyzed data includes demographics, indication, procedure type, operative times, conversions, intra- and postoperative complications, length of stay, and patient-reported satisfaction at discharge and ≥6 months postoperatively.

**Results:**
40 children underwent robotic-assisted surgery using the *da Vinci Xi System*. Included procedures are: cholecystectomy (n=10), splenectomy (n=10), intestinal resections (n=6), orchidopexy / orchiectomy (n=4), gastric/gastrostomy (n=5), diaphragmatic repair (n=2), and others (n=5). Three conversions with enlargement of the umbilical port site into a minilaparotomy were required, all for intestinal procedures, due to anatomical constraints. No increased conversion or complication rates were observed in younger or smaller children compared with older patients. The youngest patient was 16 months old with a body weight of 6.9 kg. The median age of the children at the time of the surgery was 161 months and the median weight was 45kg. Over all procedures the mean operative time was 138 min. The mean length of the hospital stay was 5 days. Patient satisfaction was high and remained stable at follow-up.

**Conclusion:**
Robotic-assisted pediatric visceral surgery appears technically feasible and safe across a wide spectrum of visceral procedures. In this cohort, the need for conversions was associated with individual anatomical factors rather than system limitations or patient age and size.

DE GRUYTER BRILL

**Another source of near-fatal bleeding - late-onset hematothorax six weeks after uneventful MIRPE**
ID: E-DCK-468

**Chirurgie (auch interdisziplinär)/ Surgery (also interdisciplinary)**
**Thorakale Prozesse / Thoracic Conditions**

Kai-Uwe Kleitsch[1], Thomas Krebs[1], Frank-Martin Häcker[1]

[1]Ostschweizer Kinderspital, Fachbereich Kinder- und Jugendchirurgie, Chest Wall Unit, St. Gallen, Schweiz

**Background:**
The minimal invasive repair of pectus excavatum (MIRPE) and its' modifications represent the gold standard for surgical repair of chest wall deformities. Severe complications during and/or after MIRPE are rare but also underreported in the literature.

**Material and methods:**
We report on a 14-year-old adolescent who underwent an uneventful repair of his asymmetric funnel chest. 6 weeks postoperatively the patient presented with shortness of breath and dizziness. Clinical examination and imaging showed a spontaneous massive right-sided hemothorax. After chest tube placement and initial drainage of 600ml bloody pleural effusion, Angio-CT imaging was performed without evidence of active bleeding, but suspicion of a lesion to the right-sided internal mammary artery. After evacuation of 1600ml bloody effusion with the patient still in stable condition, the chest tube was removed within 24 hours. The further clinical course was uneventful.

**Results:**
Accidental lesion of the internal mammary or intercostal vessels during pectus surgery represent a rare but potentially serious complication. Only a few cases with delayed and near-fatal bleeding as a complication after MIRPE are reported in the literature, many of them never demonstrated a clear source of bleeding.

**Conclusion:**
Watchful waiting was finally successful in our case. However, immediate intervention (IMA coiling by interventional radiologist or thoracoscopic surgical repair) is mandatory in case of persistent bleeding. Pectus surgeons have to be aware of this possible risk and therefore we stress the need to report on such rare complications.

DE GRUYTER BRILL

### Thermodermography as useful tool to assess effects/side effects of thoracal cryoablation after MIRPE

ID: E-DCK-494

**Chirurgie (auch interdisziplinär)/ Surgery (also interdisciplinary)**
**Thorakale Prozesse / Thoracic Conditions**

Frank-Martin Häcker[1], Philip Julian Broser[1], Kai-Uwe Kleitsch[1]

[1]Ostschweizer Kinderspital, Fachbereich Kinder- und Jugendchirurgie, Chest Wall Unit, St. Gallen, Schweiz

**Background:**
Surgical repair of chest wall deformities is associated with significant pain, and efforts to control pain impact resource utilization. Efficient pain management is a crucial and mandatory measure to ensure a good outcome and to reduce hospital stay. Vice versa, poor pain control not only increases hospital stay, but also can raise opioid consumption, limit mobility and/or favour readmissions. Within the last couple of years, an increasing number of authors reported on their experience with cryoablation for pain management. However, applicants have to be aware of possible side effects such as neuropathic pain and/or long-term sensory function impairment. In a pilot study, we started to include thermodermography as a routine measure to assess intermediate and possible long-term side effects after thoracal cryoablation.

**Material and methods:**
The regulation of blood flow and temperature control on the skin of the thorax is controlled by the intercostal nerves.  Temperature control may be used as a confidential marker to assess the vitality of the intercostal nerves. We analyze the step by step recovery of these nerves by using thermal skin imaging in our postoperative outpatient clinic follow-up program.

**Results:**
We report our preliminary experience with the first couple of patients after MIRPE. Please see attached our preliminary experience with base line imaging (left), imaging after heat application (middle) and after cold application (right)

DE GRUYTER BRILL

**Results**

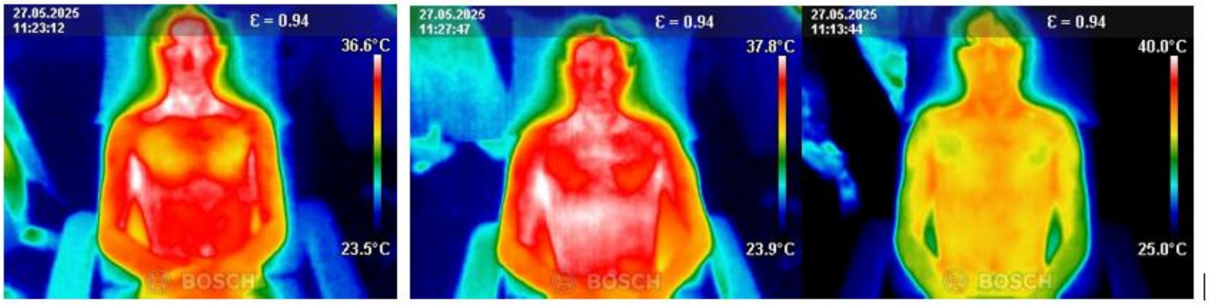

Base Line imaging after heat application after cold application

**Conclusion:**
We consider thermodermography as an additional useful tool and would like to present our preliminary results with this new assessment method.

DE GRUYTER BRILL

**20 years clinical application of the vacuum bell for conservative treatmentof pectus excavatum**
ID: E-DCK-497

**Chirurgie (auch interdisziplinär)/ Surgery (also interdisciplinary)**
**Thorakale Prozesse / Thoracic Conditions**

Frank-Martin Häcker[1]

[1]Ostschweizer Kinderspital, Fachbereich Kinder- und Jugendchirurgie, Chest Wall Unit, St. Gallen, Schweiz

**Background:**
For decades, open surgical repair was the only available method to treat Pectus excavatum (PE). In 1998 Donald Nuss described first time a minimally invasive technique for surgical repair of PE (MIRPE), and today MIRPE is performed with increasing frequency worldwide. However, despite it’s minimally invasive approach, with the widespread use of MIRPE the character and number of complications have increased. 20 years ago, non-surgical measures such as vacuum bell therapy (VBT) were introduced as a useful complement for specific treatment of PE patients.

**Material and methods:**
The study includes the results of an online survey submitted to the members of CWIG (Chest Wall International Group), a selective review of the English spoken current literature with focus on VBT and an analysis of our own previous studies concerning VBT.

**Results:**
72% of the CWIG members practicing in 47 different institutions confirmed to use VBT for PE patients. Furthermore, within the last 10 to 15 years an increasing number of studies were identified reporting on successful use of VBT for PE. However, a recently published study stated that the effect of VBT is predominantly because of thickening of the chest wall by increasing pre-sternal adipose tissue. There was one study comparing VBT to MIRPE but no randomized and/or prospective studies comparing conservative treatment vs. surgical repair or conservative treatment vs. no specific therapy. Variables predictive of an excellent outcome could be identified. Especially in younger PE patients, VBT is reported with increasing frequency in the survey as well as in the literature.

**Conclusion:**
Non-operative treatment of PE with VBT proved to be safe and a potential alternative to surgical repair in carefully selected PE patients. Patient’s age at diagnosis and severity of the PE represent relevant variables to decide which kind of therapy might be successful to correct PE. Especially in PE patients under the age of 10 years, VBT seems to represent the first step of specific therapy.

**Sclerotherapy for the management of venous malformations**
ID: E-DCK-451

**Chirurgie (auch interdisziplinär)/ Surgery (also interdisciplinary)**
**Vaskuläre Malformationen / Vascular Malformations**

Mohammad Tareq Rahimi[1]

[1]French Medical Institute for Mothers and Children (FMIC), Kabul, Afghanistan

**Background:**
Vascular malformation occurs due to different intrauterine developmental abnormalities, which result in anomalous angiogenesis involving the vascular and lymphatic system. According to Mullikin and Glowacki classification, vascular abnormalities can be broadly classified as hemangiomas which are benign vascular lesions with a proliferative and involution phase, and vascular malformations involving arterial, venous, capillary, and lymphatic tissue as well as fistulae.
The head and neck are involved in two-third of cases causing cosmetic and functional aberration. The abnormality remains silent until adolescent age when the lesion evolves into an expansile mass in response to the hormonal changes. Color Doppler ultrasound, CT scans, MRI, and angiography may be utilized to evaluate the extent of the disease. Sclerotherapy is the mainstay of treatment with absolute alcohol and sodium tetradecyl sulfate being the most commonly used sclerosing agents. Surgery is indicated only in those cases where residual lesions remain after sclerotherapy or for aesthetic purposes. Alternatively, laser therapy can be useful especially in superficial venous malformations and in oromucosal lesions. In this study we present three patients who presented with venous malformations (VMs) involving the lip and requiring 98% ethanol injection as sclerosing agent.

**Material and methods:**
**Case Presentaions:**
**Case number 1:** A 6-year-old girl, presented with lower lip mass that resulted in significant deformity of the lower lip. Doppler ultrasound study revealed well-defined hypoechoic mass of heterogeneous echotexture with multiple cystic spaces within the lesion.
**Case number 2:** A 17-year-old boy, presented with a mass in the right half of the upper lip that resulted in significant deformity and disfiguring of the upper lip.
**Case number 3:** 17-year-old, girl, presented with purple-brown colored exophytic lesion of the upper lip. CT scan revealed a well-defined soft tissue mass involving the skin and subcutaneous tissues in the medial aspect of left cheek could be representing a vascular malformation.

**Results:**
N/A

**Conclusion:**
For the VMs affecting the lips, sclerosing therapy using 98% ethyl alcohol as the sclerosing agent is a convenient and efficient modality for the management of VMs, especially in a lower socio-economical setting. As in the cases described here, this method is having good aesthetic outcomes along with preservation of function.

DE GRUYTER BRILL

**Collagen-elastin scaffold with meshgraft for trauma wounds: a seven years retrospective cohort study**
ID: E-DCK-510

**Chirurgie (auch interdisziplinär)/ Surgery (also interdisciplinary)**
**Weichteilinfektion / Soft Tissue Infections**

Gerrolt Jukema[1], Maximilian Lempert[1], Sacha Halvachizadeh[1], Hans-Christoph Pape[1]

[1]University Hospital Zurich, Department of Trauma Surgery, Zurich, Schweiz

**Background:**
Full thickness traumatic soft tissue defects are difficult to treat. Mesh graft transplantation alone causes frequently instable scar ulcerations and the need for revision surgery. For that reason, in our study we used a collagen-elastin scaffold combined with mesh graft to improve wound healing and to reduce scar ulcerations and revision surgery.

**Material and methods:**
In the period 3.2013 – 3.2020,45 trauma wounds (44 affecting lower extremities(97.7%) from different types of injuries in 43 patients (age 55.0 +- 18.2 years, 46.7% female) were treated with a collagen-elastin scaffold (Matriderm®, MD) augmented mesh graft. The wounds were separated in three subgroups (soft tissue injury (ST), closed fracture with soft tissue injury (F) and open fracture (OF). All wounds underwent surgical debridement. In 43 of 45 cases (95,5%) negative pressure wound therapy (NPWT) was applied before mesh grafting was performed. Outcome variables were, recurrence of wound defects with revision surgery and reduction of bacterial strains together with length of hospital stay and length of NPWT. For statistical analysis Student's t-test, ANOVA, Mann-Whitney U test, and Pearson's chi-squared test were used.

**Results:**
The duration NPWT therapy for preconditioning the wounds, significantly differed between the groups (F: 10.8 days; OF 22.7 days; ST: 12.6 days ($p<0.05$)). With NPWT a clinically significant reduction of bacterial burden was achieved (bacterial shift (mean (SD), F: -2.25 (1.89); OF: -1.9 (1.37); ST:-2.6 (2.2)). There was a significant difference for the number of NWPT dressing changes (OF: 4.9, F: 2.5, ST: 2,9) and length of hospital stay (OF: 48.2 days, ST: 43.5 days, F: 29,5 days) between all groups (both $P<0,05$). For the rate of recurrence there was no significant difference between the three groups (F: 0%; OF: 11.1%; ST: 9.5%).

**Conclusion:**
Augmentation of mesh split-thickness graft with a collagen-elastin scaffold (MD) is a appropriate option for full thickness wounds with take rates in our study of about 90% and improves the wound healing of trauma soft tissue defects. Especially in elderly patients with a bacterial burden in soft tissue injuries, NPWT leads to a relevant reduction of bioburden and plays therefore an important part in preconditioning full thickness wounds. After preconditioning wounds, in our study a collagen-elastin scaffold in combination with mesh grafting improves wound healing of full thickness wounds and a low number for revision surgeries was shown.

DE GRUYTER BRILL

**BEAT - Bedside Education and Teaching**
ID: E-DCK-11

**Chirurgie (auch interdisziplinär)/ Surgery (also interdisciplinary)**
**Weiterbildung und Training / Continuing Education and Training**

Nader El-Sourani[1], Marc Hofschneider[1], Ibrahim Büdeyri[1], Jens Hölzen[1], Andreas Pascher[1]

[1]Universitätsklinikum Münster, Klinik für Allgemein-, Viszeral - und Transplantationschirurgie, Münster, Deutschland

**Background:**
Medical education, particularly in surgical specialties, faces significant challenges in coordinating practical bedside teaching between faculty and students. Traditional scheduling methods rely on analog processes, leading to inefficient resource allocation, inadequate documentation of teaching hours, and limited quality control mechanisms. With increasing demands for structured medical education and the digitalization of healthcare, there is an urgent need for innovative solutions to optimize clinical teaching processes. Current systems lack integrated feedback mechanisms and fail to provide comprehensive oversight of educational activities across different training phases.

**Material and methods:**
We developed BEAT (Bedside Education and Teaching), a comprehensive digital platform designed to streamline the connection between medical educators and students across all phases of medical training. The platform was designed using modern web technologies and focuses on three core user groups: medical students (clinical semesters, rotations, and final year), faculty members (physicians and residents), and educational coordinators. Key functionalities include real-time appointment scheduling, digital logbook integration with EPA (Entrustable Professional Activities) tracking, bidirectional feedback systems, automated documentation of teaching hours, and institution-wide statistical dashboards for quality management.

**Results:**
Between June and September 2025, a total of 35 teaching sessions were offered within one surgical department, with an average attendance rate of 95%. In this period, 29 students and 4 faculty tutors actively participated. The integrated feedback system yielded high ratings: students rated teaching quality with mean scores of 5.0 (clarity of explanations), 4.9 (perceived learning gain), and 4.9 (learning atmosphere). Faculty members provided structured feedback to students through Mini-CEX assessments, with an average rating of 4.2. These results suggest high acceptance and engagement with the platform, while enabling systematic tracking of participation and feedback

**Conclusion:**
BEAT may offer a comprehensive digital solution for modernizing medical education coordination, particularly relevant for surgical training programs. By integrating scheduling, documentation, feedback, and analytics in a single platform, it addresses longstanding challenges in medical education administration. Early pilot data demonstrate high utilization, strong user satisfaction, and feasibility of implementing bidirectional feedback. The platform's focus on user experience, regulatory compliance, and educational quality makes it a valuable tool for medical institutions seeking to enhance their training programs. Future developments will focus on expanding functionality and conducting longitudinal studies to measure educational outcomes and long-term impact on medical training quality.

DE GRUYTER BRILL

**From Tradition to Innovation: Generative AI to Improve PBL Outcomes in Medical Training**
ID: E-DCK-139

**Chirurgie (auch interdisziplinär)/ Surgery (also interdisciplinary)**
**Weiterbildung und Training / Continuing Education and Training**

Rauf Safarov[1], Frank Meyer[1], Jens Wippermann[1], Max Wacker[1]

[1]Otto-von-Guericke-Universität mit Universitätsklinikum, Klinik für Allgemein-, Viszeral-, Gefäß- und Transplantationschirurgie, Magdeburg, Deutschland

**Background:**
Problem-Based Learning (PBL) is a well-established approach in medical education. In small groups, students work on real clinical cases with a high level of self-responsibility. Rooted in constructivist learning theory and the Socratic method, PBL promotes active knowledge construction, clinical reasoning, self-directed learning, and motivation. Instructors act as facilitators, guiding the group without dominating, which fosters independent learning and peer collaboration.

**Material and methods:**
Representative overview including novel conceptual ideas on PBL using the options of AI

**Results:**
(corner points)**:**
Extensive evidence shows that PBL is particularly effective in enhancing clinical skills, critical thinking, and student satisfaction. However, its impact on theoretical knowledge remains mixed. Research suggests that PBL is more effective for advanced students who already possess a foundational knowledge base and are capable of self-directed learning. This highlights the importance of aligning PBL with the learner’s developmental stage through thoughtful curriculum design.
The rapid advancement of artificial intelligence offers new opportunities to enhance the PBL model. AI-driven tools have the potential to support personalized learning, provide real-time feedback, and simulate complex clinical scenarios—bringing PBL to a new level of effectiveness and adaptability in modern medical education.
Another significant potential of generative AI, particularly when combined with effective prompt engineering, lies in its ability to efficiently generate realistic clinical scenarios. This can greatly support tutors by reducing the time and effort required to design complex case studies. AI-generated scenarios can be reviewed and refined by educators, ultimately leading to a diverse and adaptable series of clinical cases.
Moreover, generative AI offers the possibility not only to create the textual content of clinical cases, but also to enhance them through multimedia elements. By incorporating dynamic visuals, audio, and video materials, AI can help create immersive and engaging learning environments. This makes clinical case discussions more realistic, interactive, and stimulating for students—further enriching the PBL experience.

**Conclusion:**

The combination of established teaching methods such as Problem-Based Learning (PBL) with the innovations of generative AI opens up new possibilities for a modern era of medical education. While human mentoring and tutoring remain essential, the integration of AI can enhance the PBL approach and make it more engaging and dynamic for students.

Further medical education research—especially prospective studies—is necessary to test this hypothesis and explore how AI-supported PBL can be developed and implemented effectively. Such research could pave the way for taking the PBL concept to the next level.

DE GRUYTER BRILL

**Level up fracture understanding: Virtual Reality for training AO classification**
ID: E-DCK-173

**Chirurgie (auch interdisziplinär)/ Surgery (also interdisciplinary)**
**Weiterbildung und Training / Continuing Education and Training**

Vincent K. Schenk[1 2], Abdulrahman Al-Harazi[3], Hans Christian Schmidt[3], Maximilian Menger[2], Christof K. Audretsch[2], Steven Herath[2], Tina Histing[2], Markus Küper[2]

[1]Eberhard Karls Universität Tübingen, BG Unfallklinik, Tübingen, Deutschland, [2]BG Unfallklinik Tübingen, Unfall- und Wiederherstellungschirurgie, Tübingen, Deutschland, [3]SimLab - Students for Surgery, Universitätsklinikum Hamburg-Eppendorf, Hamburg, Deutschland

**Background:**
Virtual Reality (VR) describes an only artificial experience. The user moves and interacts with the virtual surrounding [1]. VR can be used for training young surgeons. Especially since decreasing resident work hours and increasing costs for training, simulation became an important tool in training of aspiring physicians. Anatomical structures can be presented in detail and modified for the need of the application, offering endless opportunities. Not only three-dimensional (3D) visualization can be achieved, but also surgical skills through all fields can be trained using VR [2].
The AO-classification is an internationally used classification for fractures of all kinds. The proper classification is important for a correct treatment and therefore good clinical outcome for the patient. Even though the AO-classification is broadly used in the clinical practice, it only score moderate inter-observer reliability, especially if subgroups are added [3]. Compared to experienced surgeons, specifically for young residents, additional 3D imaging seems to be helpful for correct classification [4]. Furthermore, it was shown that the use of a 3D classification system can increase inter-observer reliability to $\kappa=0.70$ from $\kappa=0.20$ only using 2D AO-classification [5].

**Material and methods:**
Unity® (Version 2021.3.4f1 LTS), an open-source game engine, was used for development. The program can be run und every major VR glasses. In this case an HTC® VIVE Pro and a Meta® Quest 2 were utilized. Both goggles use a head-mounted-display and can be operated by two hand controllers. The HTC® Vive Pro relies on an VR-ready computer whereas the Meta Quest 2 works independently. In this case an Alienware m15 R4 was utilized. Different types of common fractures were implemented into the program. The anonymized DICOM files of the fractures were acquired from the local trauma center. The files were stored in the “Orthanc DICOM server”. “Orthanc” allows the user to store and organize DICOM files online and access them via the internet. To make the scans available for the program, the computer storing the library and the VR google need to be in the same WIFI network. The scans will be downloaded temporarily by the VR program and deleted instantly if not needed to increase performance.
Segmentation was done utilizing Slicer3D [6, 7].

**Results:**
A VR training environment could be built. In-game, the user can move around freely and interact with the surrounding. Before viewing fractures, some basic information about the AO-classification can be observed. Afterwards the user can continue by choosing different fractures. Different fractures can be selected by clicking onto a supported bone of a 3D skeleton. Depending on the bone, different fractures will be displayed. A virtual CT scanner will be shown as well as an 3D model of the fracture. The user can interact with the 3D model and inspect the fracture from each angle. In the virtual CT viewer, the corresponding CT scan can be visualized in each of the three slice planes. The currently shown section of the CT scan can be displayed in the 3D model as semi-transparent plane. Thereby the understanding of the slice plane and the localization of the fracture, as well as the transfer from a 2D image into a 3D model can be achieved. The soft tissue of the fractures can be displayed if the localization of the fracture related to the skin surface needs to be illustrated. The program can be extended easily by adding additional fractures to the online library.

**Conclusion:**
The development of a simple training tool for correct classification of fractures could be established. The tool allows the understanding of fracture lines in 3D models and their corresponding CT scans. In the future the tool could be extended with a quiz to improve the learning experience.

**Literature:**

1. Vince, J., *Introduction to virtual reality*. 2004: Springer Science & Business Media.
2. Li, L., et al., *Application of virtual reality technology in clinical medicine.* Am J Transl Res, 2017. **9**(9): p. 3867-3880.
3. Chan, G., et al., *Inter- and intra-observer reliability of the new AO/OTA classification of proximal femur fractures.*Injury, 2021. **52**(6): p. 1434-1437.
4. Cho, Y.-c., et al., *Three-dimensional CT Improves the Reproducibility of Stability Evaluation for Intertrochanteric Fractures.* Orthopaedic Surgery, 2018. **10**(3): p. 212-217.
5. Wada, K., et al., *Intra- and inter-rater reliability of a three-dimensional classification system for intertrochanteric fracture using computed tomography.* Injury, 2020. **51**(11): p. 2682-2685.
6. Fedorov, A., et al., *3D Slicer as an image computing platform for the Quantitative Imaging Network.* Magnetic resonance imaging, 2012. **30**(9): p. 1323-1341.
7. Slicer Corporation. *3D Slicer*. 2020; Available from: .

DE GRUYTER BRILL

**Didactics as a Driver Teaching Methods and Their Impact on Interest in Surgical Training**
ID: E-DCK-257

**Chirurgie (auch interdisziplinär)/ Surgery (also interdisciplinary)**
**Weiterbildung und Training / Continuing Education and Training**

Lazaros Milonidis[1], Marko Kornmann[1]

[1]Universitätsklinik Ulm, Allgemein- und Viszeralchirurgie, Ulm, Deutschland

**Background:**
Since the summer semester 2024, a curricular OR course has been offered in Ulm for 5th and 6th semester medical students, where basic skills for daily surgical practice, such as surgical hand disinfection, are taught. As preparation, students received a structured video. Afterwards, the skills were practiced in the simulation unit of the Ulm training hospital with trained tutors. The course concluded with a debriefing by operating room nurses (OTAs), who had observed the session through one-way glass. Previous surveys during these semesters indicated only limited interest in pursuing surgical training. This study investigates the effects of different didactic formats (video, course, combination of both, or clinical clerkship alone) on preparation, interest, and career intentions in surgery.

**Material and methods:**
The video, course, and combination groups included students from the 5th/6th semester of Ulm Medical School. These students rated three Likert items (1–5): “felt well prepared,” “interest in surgery,” and “interest in a clinical elective (Famulatur).” The comparison group without video or course consisted of 8th/9th semester students who had completed a three-week surgical clerkship at the time of the survey. Career intention was assessed in all four groups (response options: abstain, yes, no, maybe). Statistical analysis was performed using the Mann-Whitney U test (effect size $r=|Z|/\sqrt{N}$) and Chi² test (Cramér’s V) for career intention. The significance level was set at $p<0.05$.

**Results:**
*Video vs. Course:* Preparation was rated higher after video ($p=0.003$; $r=0.169$). Interest in surgery was higher after the course (Surgery: $p=0.041$; $r=0.116$; Clinical elective: $p=0.007$; $r=0.151$).
*Video+Course vs. Course:* Better preparation was reported after video+course ($p<0.001$; $r=0.246$); no significant difference for either interest item ($p=0.326$/$p=0.312$; $r\approx0.06$).
*Video+Course vs. Video:* Perceived preparation did not differ significantly ($p=0.117$; $r=0.105$); however, interest was higher after video+course (Surgery: $p=0.007$; $r=0.181$; Clinical elective: $p<0.001$; $r=0.221$).
*Career intention (4×4 incl. 0):* Distributions differed significantly ($\chi^2(9)=43.572$; $p<0.001$; $V=0.169$). “Yes” was most frequently reported in the clerkship group (36.7%), followed by video+course (24.5%), course (17.3%), and video (14.9%).

**Conclusion:**
Video pre-briefings primarily strengthen the sense of preparation, while hands-on courses promote greater interest in surgery. The combination of video+course integrates both advantages: compared to video alone, students showed greater interest; compared to a structured course, they felt better prepared. For recruitment of future surgeons and readiness for surgical practice, video elements should precede and hands-on courses for key skills should be embedded in the curriculum. A blended-learning approach thus appears to be the most effective didactic strategy.

DE GRUYTER BRILL

**To be or not to be a surgeon. Students' perception of pursuing surgery as residency of choice**
ID: E-DCK-266

**Chirurgie (auch interdisziplinär)/ Surgery (also interdisciplinary)**
**Weiterbildung und Training / Continuing Education and Training**

Jean-Paul Bereuter[1], Mark Enrik Geissler[1], Rona Berit Geissler[1], Grit Krause-Jüttler[1], Jürgen Weitz[1], Marius Distler[1], Florian Oehme[1], Tobias Huber[2], Felix von Bechtolsheim[1]

[1]Department of Visceral, Thoracic and Vascular Surgery, Faculty of Medicine and University Hospital Carl Gustav Carus, TUD Dresden University of Technology, Dresden, Deutschland, [2]Department of General, Visceral and Transplant Surgery, University Medical Center of the Johannes Gutenberg-University Mainz, Mainz, Deutschland

**Background:**
Over the past decade, Germany has experienced an increasingly critical shortage of surgeons. Interest in surgical careers among medical students has declined, accompanied by a continued decrease in applications for general surgery residency positions (Kleinert et al., 2017; Petty et al., 2025; Heimke et al., 2025). Concurrently, the surgical workforce is undergoing demographic shifts: the proportion of surgeons aged over 60 years increased from 10.7% in 2015 to 14.1% in 2021, and projections suggest that by 2030 nearly one in four specialist surgical positions will remain vacant. This development is further compounded by demographic change in the general population, with an aging society expected to substantially increase the demand for surgical care (Heimke et al., 2025). Collectively, these trends highlight the urgent necessity of developing new strategies to recruit and retain medical students for surgical specialties. The design of such strategies requires a detailed understanding of the perspectives, motivations, and expectations of the current generation of medical students. The present study aims to provide a comprehensive assessment of the factors influencing their interest in pursuing surgical careers in Germany.

**Material and methods:**
This study was conducted by an interdisciplinary team of medical students and surgeons. The survey was created using LimeSurvey and distributed online between June and July 2024. Distribution channels included emails to student representative bodies at all German medical universities, as well as dissemination via chat groups, email lists, and social media platforms. Participants were additionally encouraged to share the survey with peers to maximize reach. The questionnaire consisted of 41 items and collected information on demographic characteristics, engagement with surgical disciplines, and factors influencing residency choices and broader career expectations.

**Results:**
A total of 1,059 students completed the survey, representing 33 German medical faculties. The largest proportions of respondents came from Dresden (12.9%), Heidelberg (11.1%), Hamburg (8.2%), and Halle-Wittenberg (8.2%). The majority were female (70.3%), with a mean age of 24.6 years. For the purposes of this study, "surgical specialty" contained the entities of general, vascular, cardiac, pediatric, orofacial, orthopedic, trauma, plastic, thoracic, visceral, and neurosurgery. Surgical interest was assessed on a 5-point Likert scale. Overall, 50.7% reported high interest (ratings 4–5). However, the proportion of students intending to pursue a surgical career declined from 41.5% in the first to 31% in the final semester.

DE GRUYTER BRILL

Comparison of students with low to moderate interest (ratings 1–3, n = 550) and those with high interest (ratings 4–5, n = 509) revealed marked differences in extracurricular engagement. Highly interested students were significantly more likely to be members of surgical societies (6.9% vs. 0.2%, $p < 0.001$), to attend training sessions (19.7% vs. 6.2%, $p < 0.001$), and to participate in congresses (19.8% vs. 5.5%, $p < 0.001$). They also reported greater surgical exposure, measured both in duration (median 5–8 vs. 1–4 weeks, $p < 0.001$) and the number of procedures attended ($p < 0.001$).
To identify predictors of surgical career choice, logistic regression analyses were performed. In univariate analyses, older age, advanced academic year, lecture attendance, and concerns regarding work–life balance were associated with reduced likelihood of pursuing surgery. Multivariate analysis confirmed academic year as a negative predictor (OR = 0.68, 95% CI: 0.53–0.87, p = 0.002), indicating declining interest over the course of training. Strong preference for work–family balance (OR = 0.07, 95% CI: 0.04–0.13, $p < 0.001$) and concerns about work–life balance (OR = 0.76, 95% CI: 0.58–0.98, p = 0.04) also remained significant deterrents. Conversely, high interest in surgery (OR = 3.44, 95% CI: 2.5–4.37, $p < 0.001$), attendance at surgical congresses (OR = 2.89, 95% CI: 1.18–7.05, p = 0.02), greater surgical exposure (OR = 1.34, 95% CI: 1.1–1.64, p = 0.004), elective participation in a surgical track during the practical year (OR = 3.8, 95% CI: 1.13–12.8, p = 0.03), and preference for practical work (OR = 4.55, 95% CI: 2.55–8.12, $p < 0.001$) were independently associated with surgical career intention.
A subcohort analysis further examined students who shifted toward a surgical career during medical school. In multivariate analysis, interest in surgery (OR = 3.83, 95% CI: 2.05–7.16, $p < 0.001$), surgical exposure (OR = 1.65, 95% CI: 1.18–2.31, p = 0.004), extracurricular surgical education (OR = 1.68, 95% CI: 1.14–2.46, p = 0.008), and practical orientation (OR = 6.25, 95% CI: 2.13–18.36, $p < 0.001$) were significant positive predictors of such a shift. In contrast, prioritizing work–family balance (OR = 0.04, 95% CI: 0.01–0.14, $p < 0.001$) and participation in academic programs (OR = 0.64, 95% CI: 0.42–0.98, p = 0.04) were negatively associated.

**Conclusion:**
In conclusion, our study underlines the critical importance of early, immersive exposure to the surgical field, structured practical curricular and extracurricular education, and the need to address lifestyle-related concerns in sustaining medical students' interest in surgical careers. Although many German medical students begin their training with a strong inclination toward surgery, this interest often declines due to limited practical experience and persistent concerns about work-life balance. By implementing targeted educational reforms, such as increasing access to hands-on surgical experiences, fostering extracurricular education and mentorship opportunities, and promoting flexible career models, medical schools and policy makers can play an essential role in preserving student interest in surgery and mitigating the growing shortage in the surgical workforce.

**References:**
- Kleinert R et al. Generation Y and surgical residency - Passing the baton or the end of the world as we know it? Results from a survey among medical students in Germany. *PloS one.* 2017;*12*(11), e0188114.
- Petty EG et al. Supply, Distribution, and Aging of the General Surgeon Workforce by Rurality and Over Time: A National Observational Study. *Annals of surgery.* 2025;10.1097/SLA.0000000000006758
- Heimke M et al. Fostering young talents for surgery: Laparoscopy in preclinical education-Too early or exactly right?. Chirurgie (Heidelb). 2025;96(2):136-144. doi:10.1007/s00104-024-02145-3

DE GRUYTER BRILL

**Comparison of laparoscopic performance using angulating versus standard instruments**
ID: E-DCK-272

**Chirurgie (auch interdisziplinär)/ Surgery (also interdisciplinary)**
**Weiterbildung und Training / Continuing Education and Training**

Jean-Paul Bereuter[1], Maximiliano Ceron[1], Mark Enrik Geissler[1], Josefine Runge[1], Jasmin Hasanovic[1], Anne Selbmann[1], Jürgen Weitz[1], Marius Distler[1], Felix von Bechtolsheim[1], Florian Oehme[1], Jakob Dobroschke[1]

[1]Department of Visceral, Thoracic and Vascular Surgery, Faculty of Medicine and University Hospital Carl Gustav Carus, TUD Dresden University of Technology, Dresden, Deutschland

**Background:**
Minimally invasive surgery is rapidly evolving, with numerous instruments now available alongside standard laparoscopic instruments (SLIs). Among these, articulating laparoscopic instruments (ALIs) offer enhanced articulation that mimics the dexterity of robotic systems while remaining more cost-effective and accessible. However, evidence on their learning curve and performance compared with state-of-the-art laparoscopic instruments remains limited. This study aimed to address this gap within a controlled simulator setting.

**Material and methods:**
A total of 53 medical students participated in this single-center, prospective, randomized crossover trial. After baseline training, participants were allocated to either the SLI or ALI group using block randomization. Training followed a standardized, modified Fundamentals of Laparoscopic Surgery (FLS) curriculum, during which the learning curve was tracked until a predefined proficiency threshold was achieved in the peg transfer and suture and knot tasks. Subsequently, all participants completed a final exam on both tasks using the instrument assigned by randomization. At the end of the study, a crossover assessment was conducted, allowing each participant to perform both tasks with both SLI and ALI. The primary endpoint was tissue handling skill, assessed through applied force parameters. Secondary endpoints included instrument motion, task completion time, and the corresponding learning curves of these parameters.

**Results:**
Fifty-three medical students participated in this trial. After randomization, 26 participants were allocated to the SLI and 27 to the ALI group. Overall, no significant differences were found between both groups concerning age (SLI: 25 years vs. ALI: 24.7 years, $p = 0.82$), sex distribution (SLI: 50% male vs. ALI: 67% male, $p = 0.27$), dominant hand (= DH, SLI: 89% right vs. ALI: 85% right, $p = 0.99$) and academic year (SLI: 5th AY vs. ALI: 5th AY, $p = 0.79$).
Participants of the SLI group applied a significantly higher peak force during the final exam in the Peg transfer task when using ALI compared to SLI (SLI: 1.75 N vs. ALI: 2.8 N, $p < 0.001$). In contrast, participants of the ALI group showed similar values when using both types of instruments (SLI: 1.8 N vs. ALI: 2.1 N, $p = 0.22$). Additionally, participants of the ALI group applied significantly less peak force when using ALI compared to participants of the SLI group using ALI (SLI group: 2.8 N vs. ALI group: 2.1 N, $p = 0.004$). Importantly, participants of the ALI group showed comparable performance to those of the SLI group when using SLI (SLI group: 1.8 N vs. ALI group: 1.8 N, $p = 0.97$). In line with the results for the Peg transfer task, similar patterns were observed for the suture and knot task.

Regarding motion parameters, participants of both the SLI and the ALI group used significantly less total path length when using SLI compared to ALI in the Peg transfer task. Moreover, participants of both SLI and ALI group needed more time to complete the Peg transfer task when using ALI compared to SLI (SLI group: SLI: 82.5 s vs. ALI: 412 s, $p < 0.001$; ALI group: SLI: 109 s vs. ALI: 167 s, $p < 0.001$). Importantly, participants of the ALI group were significantly faster using ALI compared to participants of the SLI group (SLI group ALI: 412 s vs. ALI group ALI: 167 s, $p < 0.001$). The same pattern was observed concerning the more complex suture and knot task.

When analyzing the learning curves (LCs) for the Peg transfer task, significant differences between the SLI and the ALI groups were found. Participants in the ALI group required significantly more time across all stages of training and plateaued at higher values, as confirmed by ANOVA ($p < 0.001$). To quantify these differences, the area under the curve (AUC) was calculated for each participant. Relative AUC values, normalizing the learning progress, demonstrated that the ALI group had a significantly slower learning curve compared with the SLI group (SLI group: 0.18 vs. ALI group: 0.42, $p < 0.001$). This pattern was not limited to task time but extended to other performance parameters, like peak force (ANOVA: $p = 0.02$; Relative AUC: SLI group: 0.5 vs. ALI group: 0.59, $p = 0.03$ and path length (ANOVA: $p < 0.001$; Relative AUC: SLI group: 0.23 vs. ALI group: 0.44, $p < 0.001$).

**Conclusion:**

Although ALI use requires a longer learning curve, training with angulating instruments enhances proficiency with both articulating and standard laparoscopic instruments. Their similarity to robotic systems suggests additional translational benefits, supporting the integration of ALI into surgical training programs in order to increase overall training efficiency.

DE GRUYTER BRILL

**Impact of video gaming and playing a musical instrument on surgical skills - a systematic review**
ID: E-DCK-273

**Chirurgie (auch interdisziplinär)/ Surgery (also interdisciplinary)**
**Weiterbildung und Training / Continuing Education and Training**

Julia Hollmann[1 2], Stephanie Trum[1 2], Oliver Muensterer[1]

[1]Department of Pediatric Surgery, Dr. von Hauner Children's Hospital, LMU Medical Center, Munich, Munich, Deutschland, [2]Klinikum Dritter Orden, Klinik für Allgemein-, Visceral-, und Thoraxchirurgie, Munich, Deutschland

**Background:**
Video gaming and musical instrument practice may exert a beneficial influence on performance in robotic surgery and laparoscopy. However, the published evidence is inconclusive: Older publications may not reflect the current gaming practice and contemporary robotic platforms. Therefore, we conducted an updated systematic review of the literature in recent years, focusing on articles published in recent years.

**Material and methods:**
A systematic literature search for review articles was conducted in PubMed/MEDLINE in September 2025 using the predefined keywords "video gaming laparoscopy", "((video gaming) AND (surgery)) AND (skills)", "((playing piano) AND (surgery)) AND (skills)", "(robotic skill) AND (playing instrument)", "(laparoscopy) AND (playing instrument)", "((laparoscopy) AND (video gaming)) AND (playing instrument)". The search was restricted to publications from the past five years, and appropriate filters were applied to refine the results. Data extraction and analysis were systematically guided by a modified version of the PICOS (Population, Intervention, Comparison, Outcomes, Study design) framework to ensure methodological rigor and consistency. Both, the study designs of the included studies and the effects of gaming and musical instrument playing were thoroughly investigated.

**Results:**
We identified 8 studies (n=8) that were included in this systematic review. Studies with positive outcomes significantly outweighed the negative ones (6 vs. 2, respectively). Two studies even showed significant improvement of gaming on laparoscopic/ robotic skills, while two other studies reported that there is no influence of playing an instrument/ gaming on laparoscopic or robotic skills. No substantial heterogeneity was observed in terms of study design.

**Conclusion:**
Recent studies from the past five years suggest that video gaming and musical instrument experience may influence robotic surgical skills. While some evidence indicates video gaming might offer advantages in laparoscopy, no definitive conclusions can be made due to varying study designs, objectives, and outcomes. Video gaming could serve as a warm-up or skill acquisition tool since positive outcomes clearly overweigh negative outcomes, but a standardized protocol is necessary to clarify its impact on laparoscopic skill development and progression.

DE GRUYTER BRILL

**Medical Dramas Shape Perceptions, but Research Experience Drives Surgical Career Interest**
ID: E-DCK-328

**Chirurgie (auch interdisziplinär)/ Surgery (also interdisciplinary)**
**Weiterbildung und Training / Continuing Education and Training**

Isabella Metelmann[1], Anna Wagner[1], Matthias Steinert[1], Sebastian Krämer[1]

[1]Universitätsklinikum Leipzig, Klinik für Viszeral-, Transplantations-, Thorax- und Gefäßchirurgie, Leipzig, Deutschland

**Background:**
Medical drama series (TV series) remain highly popular, particularly among medical students. This study aimed to investigate how such media influence students’ perceptions of surgery and their interest in pursuing a surgical career.

**Material and methods:**
We conducted an anonymous, cross-sectional online survey among medical students at Leipzig University between December 2024 and March 2025 using LimeSurvey (LimeSurvey GmbH, Hamburg, Germany). A pre-validated questionnaire assessed participants' media consumption, perception of surgery, and postgraduate career preferences. The study was reviewed and approved by the university’s data protection officer and the local ethics committee (360/24-ek). Data analysis was performed using Microsoft Excel (Microsoft Corp., Redmond, WA, USA).

**Results:**
A total of 257 students responded, of whom 220 (85.6%) reported watching medical TV series. Approximately one-third (77/214; 36.0%) stated that their perception of surgery had been influenced by such media. Among these, 38.4% (28/73) expressed interest in a surgical career. The most frequently watched series were Grey’s Anatomy (52.5%), Dr. House (38.5%), and Scrubs (35.4%).
Students influenced by TV series rated surgery significantly more positively (30/62 vs. 41/122; p=0.05); however, this was not associated with a significantly higher interest in surgical training (28/59 vs. 56/139; p=0.35). A strong association with surgical interest was found in students influenced by their own doctoral research (9/10 vs. 75/188; p=0.002). In contrast, students whose perception was shaped by personal experiences of family or friends in surgery showed significantly lower interest in a surgical career (29/86 vs. 55/112; p=0.03).
No significant differences were observed in perceptions of surgery regarding work-life balance, family compatibility, prestige, or adrenaline-driven appeal between the groups.

**Conclusion:**
Medical TV series contribute to shaping the image of surgical specialties among students but are not decisive in career choice. Instead, personal academic experiences and the influence of close professional contacts play a more significant role in fostering interest in surgical careers. Understanding these influences can support more effective guidance of students in their career choices.

DE GRUYTER BRILL

### Surgical teaching of the study of human medicine at an extra-university “Hospital of Maximal Care“

ID: E-DCK-329

**Chirurgie (auch interdisziplinär)/ Surgery (also interdisciplinary)**
**Weiterbildung und Training / Continuing Education and Training**

Peter Würl[1], Frank Meyer[2]

[1]Klinikum Dessau, Klinik für Allgemein-, Viszeral- und Thoraxchirurgie, Dessau-Roßlau, Deutschland,
[2]Otto-von-Guericke-Universität mit Universitätsklinikum, Klinik für Allgemein-, Viszeral-, Gefäß- und Transplantationschirurgie, Magdeburg, Deutschland

**Background:**
Surgical teaching has developed into various directions over recent years. In addition to the novel options by establishing the so-called “SkillsLabs“, the increasing complexity and absorption of theoretical and practical education bedside play an important role to search for new ways and chances of an improved and more sufficient practical alignment. In this context, the shift of parts of education to extra-university institutions of clinical care is one of the possible ways.
*Aim*: Based on selected, topic-related references from recent scientific literature and own teaching experiences, specifics and options of academic teaching in extra-university institutions of clinical care are described during the study of human medicine.

**Material and methods:**
Narrative structured overview

**Results:**
In the light of the spectrum of surgical care, extra-university surgical departments of each level of care can be considered *per se* suitable institutions to impart, in particular, practical knowledge and first experiences in treating frequent and relevant disorders. In this context, these institutions with their specific teaching options and strengths, in particular, in practical teaching out of the spectrum of teaching approaches, activities and instruments in the curriculum of prospective physicians cannot be thought away. Among these, teaching hospitals are considered ”hot spots“ of especially qualified and audited extra-university structures, which provide - following rules - practice-oriented parts and aspects of surgical teaching in human medicine.
The optimal function of a teaching hospital and, thus, implementation of a good teaching should predict splitting of responsibility among and accepting responsibility by the players and partners. Basicly, the players at teaching hospitals are the clinical management with its structural responsibility (staff, rooms, teaching aids) and the teaching employees out of the physician and nursing group with motivation, enough time and the necessary substantial and didactic skills.
The students fulfill their part of responsibility best by a sufficient theoretical knowledge, a good motivation, empathy to the patients and colleagues as well as an adequate resilience.
From alma mater side, the responsibility is characterized by a sufficient theoretical preparation of the students, a didactic qualification of the teachings staff with continuous offers and time-wise coordination of semester plans. The alma mater’s responsibility to provide feed back’s to all partners may not be neglected.
In the most favorable case, optimal fulfillment by each steak-holder can lead to a bilateral positive “feed back“ with a resulting mutual motivation and the development of a positive motivation spiral. Despite the similar profiles of requirements and transfer of knowledge among the various learners, there are a couple differences. Beside pupils after they had graduated from school in their (so-called) “Free Social Year“, the learners are consisting of interns, seminarists of various semesters, medical students in their internships of the 4th year and 6th year in the study of human medicine.
The didactic approach and the contents overlap in part substantially from group to group. However, the use of various teaching formats is more differentiated. Known formats such as clinical examen courses, daily practice ward rounds or teaching rounds, patient presentations, problem-oriented learning (POL), simulations of examinations; participation in clinical services, doctor’s consultations, change-of-shift reports and clinical rounds; radiological case presentations, M&M conferences or interdisciplinary tumor-board conferences are used differently in the various groups.

**Conclusion:**
The methodological as well as the contentual claims of surgical teaching are steadily increasing in the light of the lack of physicians on one side and the more and more broad field of activity of the single physician on the other side. In addition, the “change of the climate“ by the more monetarily oriented health system requires a sufficiently high number and adequately educated staff. In contrast, the conditions are becoming more and more difficult to fulfill this demand. The stringent and clocked work-flow and processes in the surgical departments, the high level of work intensification, the enormous part of bureaucratic activities, lack of staff, increasing percentage of out-patient clinic care and the more strict frame concerning industrial law make it more difficult for the clinics and hospitals including the teaching hospitals to sustain and improve the required offers in surgical teaching.
ll steak-holders in the system and with responsibility know that maintenance of the quality in health care and - above all - its further development can only succeed if also extra-university institutions of health care are ready to bear the future challenges and to stand shoulder-to-shoulder with all the responsible players.
In conclusion, extra-university institutions accomplish not only as a welcome opportunity the practice-oriented part in the teaching spectrum of the study of human medicine. In the light of the ongoing transformation of the health care system, they become a more and more important part of the educational structure. To accept this structural and contentual challenge in various and variable manners is one of the main tasks in addition to clinical care for these institutions.

DE GRUYTER BRILL

### Medical and Surgical Specialty Training in Germany, Switzerland, and Italy: A Comparative Overview

ID: E-DCK-367

**Chirurgie (auch interdisziplinär)/ Surgery (also interdisciplinary)**
**Weiterbildung und Training / Continuing Education and Training**

Lorenzo Mazzucchelli[1], Frank Meyer[2], Armin Kraus[1]

[1]Otto-von-Guericke-Universität mit Universitätsklinikum, Klinik für Plastische, Ästhetische und Handchirurgie, Magdeburg, Deutschland, [2]Otto-von-Guericke-Universität mit Universitätsklinikum, Klinik für Allgemein-, Viszeral-, Gefäß- und Transplantationschirurgie, Magdeburg, Deutschland

**Background:**
Postgraduate surgical training in Europe varies widely in structure, supervision, and educational philosophy, influencing the quality and consistency of resident formation.

**Material and methods:**
(incl. Aims): This paper compares surgical specialty training in Germany, Switzerland, and Italy across several key parameters: access and selection processes, time spent in the operating room, theoretical instruction, opportunities and requirements for research, supervision and mentorship, rotation structure, training duration and flexibility, assessment and certification methods, simulation-based training, autonomy in clinical practice, work-hour regulation, and financial and contractual conditions.

**Results:**
(corner points): - In Germany and Switzerland, training access is decentralized, with candidates applying directly to hospitals through job interviews and training regulated by medical chambers or scientific societies.
- Italy, in contrast, follows a centralized system governed by the Ministry of Education, with entry based on a national competitive exam and residency conducted within university-based programs.
- Swiss training is characterized by a high degree of standardization and structured evaluation, while Germany offers greater institutional variability.
- Italian programs benefit from national curricula but often show heterogeneity in operative exposure and research integration.

**Conclusion:**
This comparative analysis highlights strengths and limitations within each system, offering perspectives on harmonizing surgical education and promoting mobility within the European training landscape.

DE GRUYTER BRILL

**Surgical video instructions via QR-codes in expert tutored training with 3D biotissue models**
ID: E-DCK-368

**Chirurgie (auch interdisziplinär)/ Surgery (also interdisciplinary)**
**Weiterbildung und Training / Continuing Education and Training**

Laetitia Hampe[1], Amila Cizmic[1], Frieda Häberle[2], Rebecca Angresius[1], Maxim De Wulf[4], Matteo Mueller[3], Stefan Bouwense[4], Jan Jonas[3], Otmar Buyne[5], Ville Sallinen[6], Stefan Breitenstein[7], Philip Müller[8], Marko Kraljevic[8], Lana Fourie[8], Stefan Holland-Cunz[9], Floris Ferenschild[5], Caroline Williamson[10], Christoph Reissfelder[11], Christiane Bruns[12], Christian Toso[13], Benjamin Weixler[7], Beat Müller[8], Pierre Clavien[3], Friedrich Herbst[14], Peter Vogelbach[15], Dieter Hahnloser[16], Stephan Bischofsberger[17], Walter Brunner[17], Alain Sauvanet[18], Sébastien Gaujoux[19], Sven Mieog[20], Stefano Partelli[21], Elena Rangelova[22], Jérôme Ammann[23], Eva Boldrini[5], Lars Vanderbroeck[4], Rainer Grotelüschen[1], Philipp Busch[1], Faik Uzunoglu[1], Anna Nießen[1], Tarik Ghadban[1], Asmus Heumann[1], Nathaniel Melling[1], Ramiz Wahib[1], Oliver Mann[1], Anna Dupree[1], Thilo Hackert[1], Harry van Goor[5], Felix Nickel[1], Anas Preukschas[1]

[1]University Medical Center Hamburg-Eppendorf, Department of General, Visceral and Thoracic Surgery, Hamburg, Deutschland, [2]Klinikum rechts der Isar, Technical University of Munich, München, Deutschland, [3]University Hospital Zurich, Zürich, Schweiz, [4]Maastricht University Medical Center, Maastricht, Niederlande, [5]Radboud University Medical Center, Nijmegen, Niederlande, [6]Helsinki University Hospital, Helsinki, Finland, [7]Cantonal Hospital Winterthur, Winterhur, Schweiz, [8]University Digestive Health Care Center Basel, Basel, Schweiz, [9]Children's University Hospital, Basel, Schweiz, [10]Skåne University Hospital, Lund, Sweden, [11]Universitätsmedizin Mannheim, Mannheim, Deutschland, [12]University Hospital of Cologne, Köln, Deutschland, [13]Geneva University Hospitals, Genf, Schweiz, [14]Medical University of Vienna, Department of General Surgery, Wien, Österreich, [15]Kantonsspital Graubünden, Chur, Schweiz, [16]University Hospital Lausanne, Lausanne, Schweiz, [17]St-Gallen Kantonsspital, St-Gallen, Schweiz, [18]Hôpital Beaujon, Paris, Frankreich, [19]GH Pitié-Salpêtrière, Paris, Frankreich, [20]Leiden University Medical Center, Leiden, Niederlande, [21]Vita-Salute San Raffaele University, Mailand, Italien, [22]Sahlgrenska University Hospital, Gothenburg, Sweden, [23]ak-medizin, Münchwilen, Schweiz

**Background:**
Complex anastomoses like the pancreato- and hepaticojejunostomy require repetitive training and time to master. Expert tutored surgical training courses to teach these anastomoses are time-limited, costly and therefore not always accessible or feasible. This study aims to evaluate the usefulness of easily accessible surgical video instructions via QR-codes in open and robotic hepatopancreaticobiliary surgery in the context of expert tutored surgical training courses.

**Material and methods:**
Trainees and tutors at the Davos Course for HPB-Surgery (April 6th – April 9th 2024), at the Hands-On-Course of the North-German Surgical Association (June 5th, 2024) and at the ESSO Advanced Course on Pancreatic Tumours Surgery (September 4th – September 5th 2025) were provided with QR-codes giving access to surgical video instructions of open and robotic pancreato- and hepaticojejunostomy with 3D biotissue organs. After watching the videos and performing the anastomoses on 3D biotissue organs participants were asked to anonymously complete an online questionnaire evaluating the videos. The data was collected using Google Form and statistics were generated using SPSS. The answers “Agree” and “Totally agree” were noted as a positive response on the Likert-scale.

**Results:**
A total of 51 participants were included in the study. The mean age was 36 years (range 27–64), with 29 males and 21 females. Participants reported an average of 8 years of surgical experience (range 0.2–35). Most participants reported that QR-codes facilitated access to training videos (n = 48, 96%) and that the videos improved comprehension of surgical steps (n= 46, 90%). The majority considered the videos with 3D biotissue organs useful (n = 31, 62%) and perceived them as an added benefit to expert tutoring. Most participants indicated that prior access to videos (n = 48, 94%) and to both videos and organ models (n = 43, 86%) would improve course preparation. Nearly all participants preferred a combination of expert tutoring and videos, than expert only (n = 49, 96%) and agreed that tutoring was more effective when supported by videos (n = 44, 86%). Most participants also agreed that they could train efficiently (n = 21, 42%) and improve their surgical skills (n = 28, 55%) using the simulated organs and training videos on their own without expert tutoring. Correlation analysis revealed that older and more experienced respondents were more likely to agree that the training videos provided an added benefit to expert tutoring ($\rho$= 0.294, p=0.042 and $\rho$=0.132, p=0.023).

**Conclusion:**
QR-code–based surgical video instructions were well accepted and perceived as valuable when integrated into expert-tutored courses on complex procedures such as pancreato- and hepaticojejunostomy. Most participants favored a combination of videos and expert tutoring, while fully self-directed training with videos without expert guidance was considered insufficient. A follow-up randomized study is underway to compare self-directed training supported by QR-code accessible videos with traditional expert tutored training.
not seen in the results section of the abstract?

DE GRUYTER BRILL

**Development and Validation of a High-fidelity Silicone Duodenal Atresia Model for Surgical Training**
ID: E-DCK-391

**Chirurgie (auch interdisziplinär)/ Surgery (also interdisciplinary)**
**Weiterbildung und Training / Continuing Education and Training**

Rebecca Angresius[1], Anas Preukschas[1], Laetitia Zoe Hampe[1], Amila Cizmic[1], Ivo de Blaauw[2], Floris Ferenschild[2], Harry van Goor[3], Christian Tomuschat[4], Stefan Holland-Cunz[5], Walter Brunner[6], Thilo Hackert[1], Felix Nickel[1]

[1]Department of General, Visceral and Thoracic Surgery, University Medical Centre Hamburg-Eppendorf, Hamburg, Germany, Hamburg, Deutschland, [2]Department of Pediatric Surgery, Radboudumc University Medical Centre, Nijmegen, Netherlands Antilles, [3]Departement of Primary and Community Care Radboud University Medical Center, Nijmegen, Netherlands Antilles, [4]Department of Pediatric Surgery, University Medical Centre Hamburg-Eppendorf, Hamburg, Germany, Hamburg, Deutschland, [5]Department of Pediatric Surgery, University Centre beider Basel, Basel, Switzerland, Basel, Schweiz, [6]Department of General Surgery, Canton Hospital of St.Gallen, Switzerland, St. Gallen, Schweiz

**Background:**
Duodenal atresia is a rare congenital anomaly requiring early surgery, but its low incidence limits pediatric surgical training opportunities[1].
Animal cadaver models are scarce, pose ethical, logistical, and hygiene challenges, and lack consistent durability. Systematic simulation-based training offers a more ethical alternative to patient-based learning, enabling novices to acquire skills effectively [2].
There is thus a need for cost-effective silicone models with realistic tissue haptics to support training in open, laparoscopic, and robotic duodenal atresia repair, particularly in settings with limited resources [3].
This study aimed to design and validate such a model, ensuring high anatomical and tactile fidelity for versatile surgical simulation.

**Material and methods:**
A silicone-based duodenal atresia simulation model was developed in a multi-step process.
First, an anatomical template of the neonatal upper gastrointestinal tract was designed according to average size and proportions using computer-aided modeling and fabricated via fused deposition modeling (FDM) 3D printing (Creality CR-10, Creality 3D Technology Co., Ltd., Shenzhen, China) with polylactic acid (PLA) filament.
The printed master mold included detailed gastric and duodenal anatomy to guide subsequent casting.
The silicone mixture was poured into the 3D-printed mold and allowed to cure at room temperature. After curing, the hollow intestinal segments were carefully extracted.
The printed mold included detailed gastric and duodenal anatomy to guide silicone casting. Flexible intestinal segments were produced by casting silicone around cylindrical rods, then integrated into a polyurethane foam abdominal base replicating the anatomical region. Pediatric surgeons of varying experience (n = 14) participating in the gastrointestinal pediatric surgery course in Davos, Switzerland, evaluated haptic realism, rigidity, anatomy, and teaching suitability for diamond-shaped anastomosis using a 5-point Likert scale (1 = completely disagree; 5 = completely agree).

**Results:**
In the initial development process the duodenal atresia model demonstrated high educational suitability. Training on the model was rated as highly valuable for teaching the diamond-shaped anastomosis (4.7 ± 0.5 on 1–5 Likert scale), with structured learning of procedural steps (4.6 ± 0.8) and strong perceived transferability of skills to the clinical setting (4.3 ± 0.6). Overall value for training was scored very high (4.6 ± 0.6), and repeating the exercise was considered beneficial (4.6 ± 0.5). The model was judged suitable for laparoscopic/robotic techniques (4.4 ± 0.7) and moderately suitable for training of open repair (3.4 ± 1.5). Visual aspects (4.0 ± 0), depth realism (3.7 ± 0.47), and size realism (3.6 ± 1.0) were rated favorable, while anatomical replication (3.5 ± 0.8), organ positioning (3.5 ± 0.7), and duodenal haptics (3.2–3.3 ± 0.9) were moderate. Haptics during suturing were scored lowest (3.0 ± 1.0). Notably, expert pediatric surgeons consistently rated the model higher than residents, particularly regarding haptic realism and suitability for anastomosis training. Session evaluation was 4.3 ± 0.5 among consultants compared to 3.9 ± 0.4 among residents; depth realism was rated 3.9 ± 0.4 versus 3.6 ± 0.5, respectively. These differences underline the model's construct validity. The model was also perceived as an appropriate replacement for animal models (3.5 ± 1.3) and provided an adequate level of difficulty (3.8 ± 0.8).

**Conclusion:**
This silicone-based duodenal atresia model offers a reproducible and low-cost platform for pediatric surgical training and reduces the reliance on animal models. It is well suited for acquiring essential and advanced skills in open, laparoscopic and robotic techniques, even in minimally equipped settings. Some anatomical and haptic limitations remain, particularly in replicating the feel of duodenal suturing, but these do not outweigh its substantial educational value. Future refinements such as thinner bowel structures may further improve realism without compromising simplicity. Building on the results of the first evaluation, targeted adaptations are currently being integrated into a second model which will undergo expert validation to strengthen anatomical fidelity, haptic realism and training applicability. Further studies should additionally assess skill acquisition as content validity and differentiate performance between residents and experts as construct validity.

1. European, C., *Prevalence charts and tables.* 2022.
2. Yokoyama, S., et al., *Evaluation methods and impact of simulation-based training in pediatric surgery: a systematic review.* Pediatr Surg Int, 2019. **35**(10): p. 1085-1094.
3. Joosten, M., I. de Blaauw, and S.M. Botden, *Validated simulation models in pediatric surgery: A review.* J Pediatr Surg, 2022. **57**(12): p. 876-886.

DE GRUYTER BRILL

**Students' perceptions towards virtual reality and phantom model training: a comparative study**
ID: E-DCK-59

**Chirurgie (auch interdisziplinär)/ Surgery (also interdisciplinary)**
**Weiterbildung und Training / Continuing Education and Training**

Juliane Kröplin[1], Constanze Friedrich[1], Lisa Harms[1], Ingo Buttchereit[1], Jan-Hendrik Lenz[1], Bernhard Frerich[1]

[1]University Medical Centre Rostock, Rostock, Deutschland

**Background:**
Virtual reality (VR) simulators are considered a promising tool for significantly enhancing surgical skills. This study aims to compare the use of a VR simulator with a conventional phantom model in oral surgery training. The findings should contribute to the further development of oral surgery simulation training and help maximize the innovative benefits of VR-based training.

**Material and methods:**
This prospective, cross-sectional, single center study analyzed students' competence in performing an osteotomy on a displaced tooth 38. Participants were dental students in their 5th semester at a University Medical Center in Germany. The study compared a VR simulator with a phantom model. After completing the exercise, students filled out a questionnaire consisting of both standardized and open-ended questions to allow for a comprehensive subjective evaluation of the training experience.

**Results:**
A total of 22 students took part in the study (12 female, 10 male). None had previously performed the exercise on a patient. From the students' perspective, both training methods were easy to learn (VR:2.0; Ph: 2.1; $p=0.642$). Individual feedback highlighted that the VR simulator's main advantages were sustainability ($n=12$) and objectivity ($n=5$). However, standardized responses indicated that the phantom model was rated as more realistic, particularly regarding anatomical representation ($p<0.001$), haptic feedback ($p<0.001$) tooth removal ($p<0.001$) and osteotomy steps ($p<0.001$). Both exercises were equally engaging for students (VR:1.8; Ph:1.5; $p=0.162$).

**Conclusion:**
Simulation-based training is an engaging way for students to learn surgical tooth removal. Currently, conventional simulation is subjectively perceived as superior to VR simulation, particularly in terms of realism. However, VR simulation allows for a more objective assessment of performance and is considered superior to the phantom model in terms of sustainability. Further technological advancements and improvements in realism could help maximize the innovative benefits of VR-based training.
